# Modeling and cleaning RNA-seq data significantly improve detection of differentially expressed genes

**DOI:** 10.1186/s12859-022-05023-z

**Published:** 2022-11-16

**Authors:** Igor V. Deyneko, Orkhan N. Mustafaev, Alexander А. Tyurin, Ksenya V. Zhukova, Alexander Varzari, Irina V. Goldenkova-Pavlova

**Affiliations:** 1 Laboratory of Functional Genomics, К.А. Timiryazev Institute of Plant Physiology RAS, Moscow, Russia 127276; 2 Genetic Resources Institute, Azerbaijan National Academy of Sciences, AZ1106 Baku, Azerbaijan; 3 Laboratory of Human Genetics, Chiril Draganiuc Institute of Phthisiopneumology, MD2025 Kishinev, Republic of Moldova

**Keywords:** RNA-seq, Data cleaning, Data filtering, De-noise, Differential expression, Statistical modeling

## Abstract

**Background:**

RNA-seq has become a standard technology to quantify mRNA. The measured values usually vary by several orders of magnitude, and while the detection of differences at high values is statistically well grounded, the significance of the differences for rare mRNAs can be weakened by the presence of biological and technical noise.

**Results:**

We have developed a method for cleaning RNA-seq data, which improves the detection of differentially expressed genes and specifically genes with low to moderate transcription. Using a data modeling approach, parameters of randomly distributed mRNA counts are identified and reads, most probably originating from technical noise, are removed. We demonstrate that the removal of this random component leads to the significant increase in the number of detected differentially expressed genes, more significant *p*values and no bias towards low-count genes.

**Conclusion:**

Application of RNAdeNoise to our RNA-seq data on polysome profiling and several published RNA-seq datasets reveals its suitability for different organisms and sequencing technologies such as Illumina and BGI, shows improved detection of differentially expressed genes, and excludes the subjective setting of thresholds for minimal RNA counts. The program, RNA-seq data, resulted gene lists and examples of use are in the supplementary data and at https://github.com/Deyneko/RNAdeNoise.

**Supplementary Information:**

The online version contains supplementary material available at 10.1186/s12859-022-05023-z.

## Introduction

**Fig. 1:**
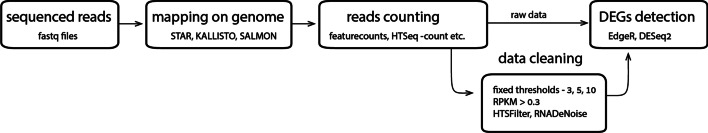
Typical workflow of a RNA-seq data analysis. Data cleaning step, although optional, is frequently included either by setting a threshold for minimal RNA counts or by more complex measures

Investigation of the mechanisms underlying differential gene expression is one of the fundamental tasks towards understanding the functional organization of genomes. Experimental quantification of gene expression is typically realized using RNA-seq technology [1]. Analysis of RNA-seq data is organized in pipelines covering many steps from trimming the sequence reads to the final detection of differentially expressed genes (DEGs) (Fig. 1). A number of programs exist for detection of DEGs, utilizing different statistical approaches focused primarily on estimating data variance [2–7]. But still other factors exist affecting statistical calculations such as noise, either technical or biological, and steps to eliminate it are also included into pipelines.

Usually noise, or in other words mRNAs with very low counts, are removed by setting some minimal threshold. Its choice is very controversial, and may vary from zero in anota2seq [5] to ten (Corset [8]) or even 32 [9] RNA reads per gene. If reads are normalized to values like FPKM/RPKM (Frequency/Reads Per Kilobase per Million reads), a threshold is similarly set to FPKM > 0.3 [10, 11]. Other ad hoc ideas include filtering genes with a total count across all experiments below a given threshold [12] or if half of the samples have counts below some threshold (this rule can be extended on multiple sample designs, DESeq2 [3] manual), filtering genes with at least one zero count in any experiment [13], or filtering according to an internal logic of a DEGs detection program [14].

Application of independent filtering of RNA-seq data, also called pre-filtering or cleaning, were shown to increase the detection power [15], and parameters of a such pre-filtering at best should be estimated using the data itself. For example, application of Jaccard index (HTSfilter), was shown to improve detection power for moderately to highly expressed genes [16]. Here we should define for clarity, the difference between filtering and normalization—filtering is a removal of values which fall under some criteria; normalization is a rescaling of values (read counts) based on some statistics.

In this work it is suggested to set up filtering, based on the statistical modeling of the read count distributions independently for each sample. Observed counts are assumed to come from two origins – real and random, and the distribution parameters of the both are fitted to the observed data. This allows the contribution of the random component to be estimated and subsequently removed from the measured expression values. We demonstrate that this approach is more effective, compared to the pre-defined thresholds, especially when searching genes with low to moderate transcription. The method is robust against sample multiplexing, does not introduce bias towards low count genes, and excludes subjectivity when setting a threshold for minimal counts. Performance was shown on our ribosome profiling data and on the other three RNA-seq datasets covering different organisms and sequencing technologies.

## Algorithm and implementation

**Fig. 2:**
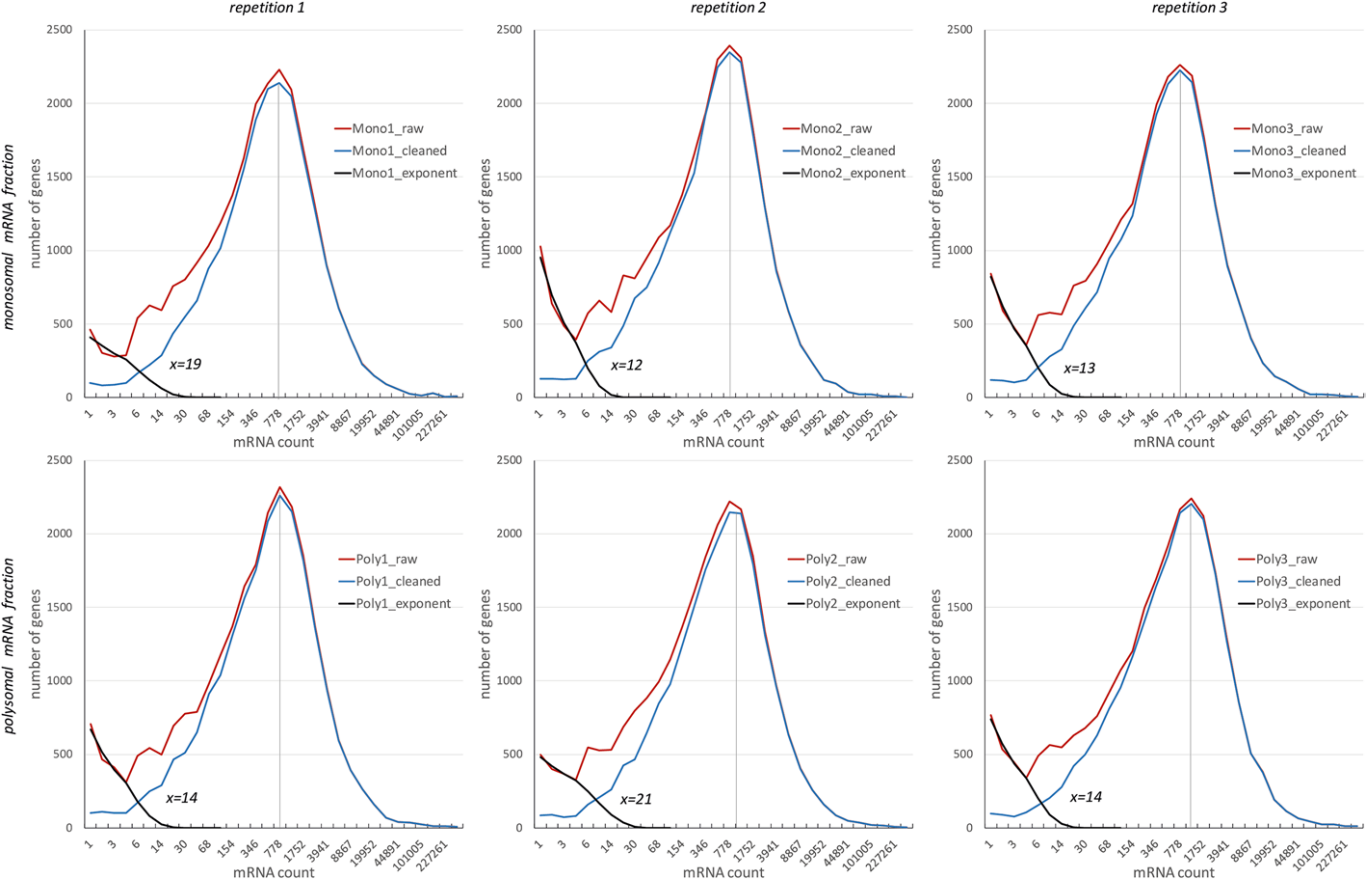
Distribution of mRNAs counts in polysomal and monosomal mRNA fractions. Figures show the number of genes with respective mRNA count for raw mRNA data (red line), cleaned data (blue) and exponentially distributed counts (black). Subtraction values *x* are calculated independently for each sample (repetition and fraction), and are defined as a 0.99 quantile of the respective exponential distribution. Distribution of cleaned data (blue line) is now very close to negative binomial

The construction and testing of the method will be done using our data on polysome profiling, which consists of three datasets representing polysome, monosome and total mRNA fractions (NCBI SRA BioProject ID PRJNA731322). Distributions of mRNA counts (Fig. 2) reveal two local maxima – at the minimum and around 780 counts. The latter can be interpreted as representing real mRNAs, while near-zero counts are assumed to originate from a random noise (either biological or technical). It is common to remove such mRNAs by setting a minimal threshold [3, 8, 9], but here we have exploited a data modelling approach to find exactly how many counts could have arisen from a random process.

To model the raw data, one can assume that the distribution curves (red curves in Fig. 2) represent a sum of two independent processes, one is exponentially distributed and the other distributed negative binomially. The former can be interpreted as a background noise, which usually decays exponentially [17], and in our case may originate from DNA debris, reverse transcription or other sequencing artifacts. The latter is a real signal that has a negative binomial distribution [7, 18, 19]. Formally, this may be represented as a sum of two independent random variables, one following a negative binomial distribution and the other an exponential:

1 $${N}_{f,i,r}={N}_{f,i,r}^{NegBinom}+{N}_{f,r}^{Exponential},$$

where *N*_*f,i,r*_ is a raw number of mRNA reads for gene *i* in mRNA fraction *f (polysome, monosome)* and repetition *r* (1, 2, 3). Of note, the exponential part ${N}_{f,r}^{Exponential}$ is assumed to vary only between mRNA fractions and repetitions.

In other words, each measured mRNA count value is assumed to consist of real and random parts. It is not possible to decompose each value into two components, but it is possible to estimate the maximum contribution of the random part and subsequently subtract it.

The probability density function of an exponential distribution can be found by fitting the exponential model into the raw data. The binomially distributed counts have a peak around 780 reads and its influence is negligible at values close to zero and therefore, points near zero are of pure random nature to which the exponent y = Ae^-αx^ can be fitted.

This can be done using lm() function in R: lm(log(y) ~ x,z), where z is a vector of the first m points of the distribution. Looking at the distributions in Fig. 2 (and  Additional file 1: Figs. S2, S4, S6), one may conclude that the first four to five points are indeed decaying exponentially. Accordingly, the first four points are used to fit the model, which already provides sufficient accuracy (black curves in Fig. 2). Finally, solving the inequation

$${\int }_{1}^{x}\text{A}{e}^{-\propto t}\text{d}\text{t}\le (1-0.01){\int }_{1}^{\infty }\text{A}{e}^{-\propto t}\text{d}\text{t} \le {\int }_{1}^{x+1}\text{A}{e}^{-\propto t}\text{d}\text{t} ,$$

for *x*, gives the point where the “tail” of the distribution is below some value, here 0.01.

Therefore, according to the above formula (1), subtracting *x* from each mRNA count will remove all random reads with 0.99 probability (CleanStrength parameter in RNAdeNoise). If for some mRNAs the resulting count value is negative, a zero value is assigned. Of note, *x* can also be defined as a point at which the absolute value of the exponent drops below a certain threshold, for example three counts. Thus, simple equation Ae^-αx^≤ 3 defines the required value of *x*. This simplified approach was implemented during the initial development of RNAdeNoise and can be used as an option.

In application to our data, modeling each dataset gives *x* values ranging from 12 to 21 (Fig. 2). So for example, to clean the repetition one of monosomal fraction the value of 19 should be subtracted from the counts for each gene. The distribution of the cleaned data is now very close to the negative binomial (blue curves in Fig. 2). The mode (most frequent value) is around 780 counts with a very small variation between datasets. In contrast, the exponential part reveals significant differences – in most samples it decreases sharply, but in two samples its contribution is more pronounced – the subtraction values reach 19 and 21. This shows that even with standardized sample preparation and sequencing routines, variation in noise levels could be significant.

The described method was implemented as a function in R [20], which is de facto a standard environment for scientific calculations, and can be found in the supplementary material (Additional file 2) and at GitHub. The function RNAdeNoise has two input parameters—a table of RNA counts organized in columns with a format identical to those used in STAR, EdgeR or DESeq2, and the filtering strength. As an output, the function returns cleaned data and subtracted values for each sample. In the following sections, the benefits of the suggested data cleaning will be exemplified using our data and three other datasets. The effect of filtering on detecting genes with different levels of expression will be investigated using a total mRNA fraction. Genes will be classified according to overall transcription into low transcribed genes (lowest 1/3 quantile of all genes, ≤ 268 counts), moderate (middle 1/3 quantile, 269…1305 counts) and highly transcribed (top 1/3 quantile, ≥ 1306 counts).

## Results

To evaluate the performance of RNAdeNoise, it has been applied to our data and other published datasets and results compared to other cleaning methods – fixed thresholds [3, 5], FPKM [21], HTSFilter [16] and samples-based filtering. Particularly, raw data were cleaned as follows: *Fixed thresholds > 3,5,10 –* if a raw read count is ≤ 3 (5, 10), then it is zeroed; *FPKM > 0.3* – if frequency per kilobase per million ≤ 0.3, then it is zeroed. HTSFilter as described in the original publication [16]. Samples-based filtering – half of the samples should have counts each above a threshold − *3,5* and *10* counts (½samples > 3,5,10). RNAdeNoise with a default stringency parameter 0.9 (R function in the Additional file 2). DEGS were identified using two programs –EdgeR [2] (default normalization TMM) and DESeq2 (built-in normalization) [3]. Criteria for DEGs if not indicated otherwise: |log2(FoldChange)|>1.5, *p-*value < 0.0001, where log2(FoldChange) and *p-*value are the output values of the respective program. In the analysis of published data criteria for DEGs was adjusted to correspond to the number of genes reported. Functional classification of genes was performed using DAVID [22].

**Fig. 3:**
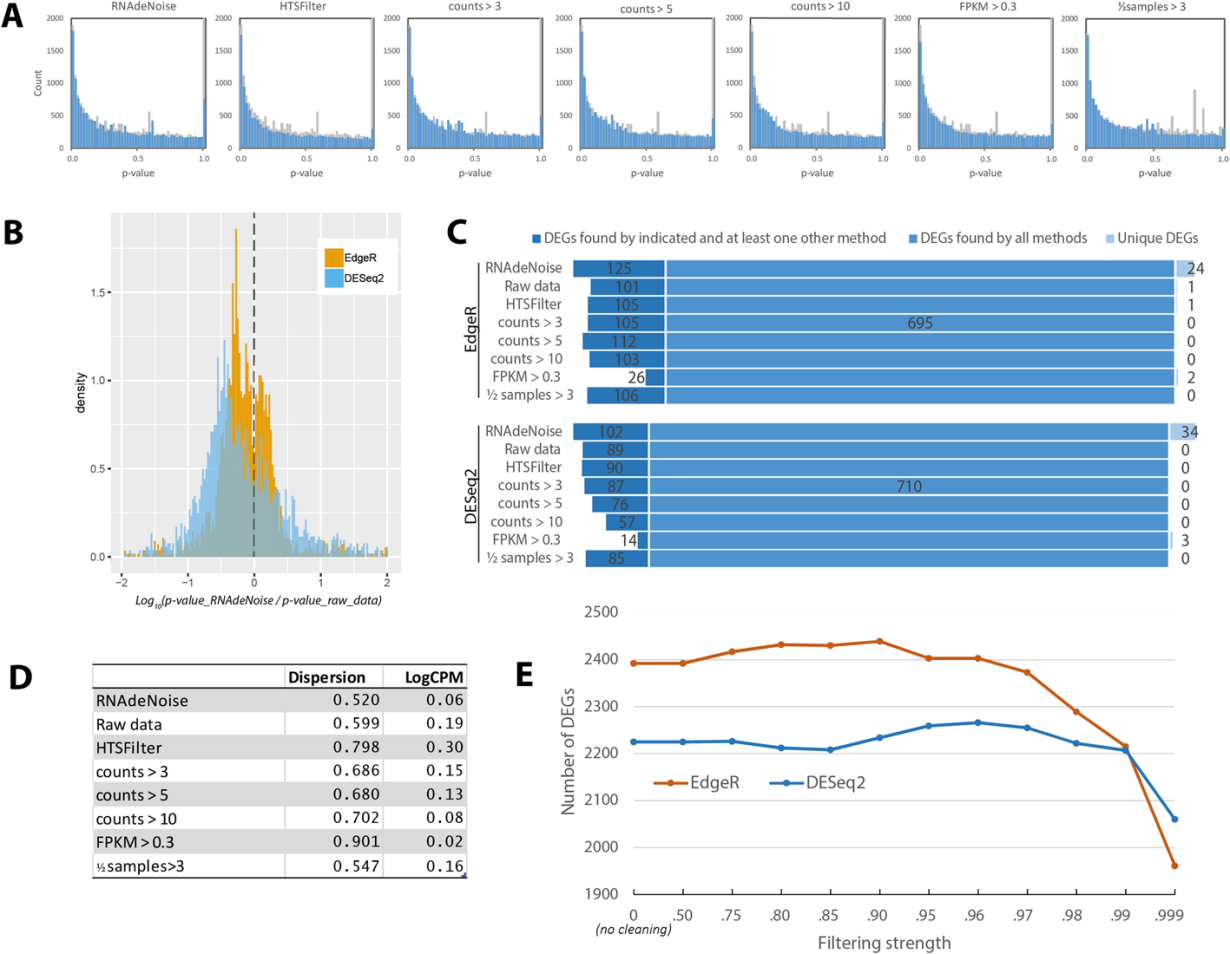
Comparison of different data cleaning procedures.** A** Histograms of *p*-values of DEGs after application of different filters. Histograms in the background (grey) represent the p-values of raw data, in foreground of filtered data (more filters are in Additional file 1: Fig. S1). **B** Histogram of ratios of *p*-values before and after cleaning with RNAdeNoise. Asymmetry against negative values shows higher significance of DEGs after cleaning. **C** DEGs identified by all filters, by two or more filters and DEGs unique to each filter. RNAdeNoise detects most of genes detected by other methods plus many new genes. Presented are genes with moderate expression on which filtering has a strongest effect (Additional file 1: Table S2). **D** Average per-gene dispersion (EdgeR parameter tagwise.dispersion) and LogCPM for raw data and data cleaned by different filters. **E** Number of detected DEGs as a function of the filtering strength

The effects of the cleaning can first be seen by the changes in the distribution of *p*-values before and after filtering [16]. All filters are capable of reducing genes contributing to a peak of *p*-values close to one (Fig. 3A; extended data in  Additional file 1: Fig. S1), and effectively minimize *p*-value discretization due to low counts. Per-gene changes in *p-*values, shows that filtering with RNAdeNoise on average increases the significance of the results (Fig. 3B). The asymmetry of the distribution against negative values shows that the cleaning generally leads to lower *p-*values of detected DEGs. For example, DESeq2 reports on average 3.16 times lower *p*-values for a gene, after the data was cleaned with RNAdeNoise. As a consequence, this translates not only to a higher number of detected DEGs, but also to the DEGs repertoire (Fig. 3C). There are core DEGs recognized by all methods, DEGs recognized by certain methods and also unique DEGs, identified solely by RNAdeNoise,  and missed by all other methods. The results of RNAdeNoise are the most inclusive – they include most of the DEGs found by other methods, plus it has 24 (34 by DESeq2) unique DEGs. Out of these 24 uniquely discovered genes there are genes like nuclear RNA polymerase D1B, growth-regulating factor 1 or methyltransferase MT-A70 protein, that are directly involved in regulating transcription (all genes are in Table S1 (Additional file 1).

We found that after filtering, a per-gene dispersion calculated by EdgeR program is reduced (Fig. 3D), which directly influences the calculation of statistical significance. This explains, why DEGs found using RNAdeNoise get more significant *p* -values and why the presented genes are missed by other methods.

The cleaning method proposed here has a single variable parameter – the filtering strength, which is a removed quantile of the exponentially distributed counts. We computed the dependency between this parameter and the number of detected DEGs (Fig. 3E). Overall, the maximum is reached at 0.9 when the program EdgeR is used, and between 0.95 and 0.97 for DESeq2. As was mentioned earlier, the filtering strength is a probability, that all random reads are removed, which implies, that some of the “real counts” may also be removed. This can be seen for the higher values of filtering strength, which leads to the removal of too much “real counts”, and hence to the sharply reduced number of DEGs. Users are supposed to set this parameter according to the program used and a desired filtering strength, but a default value of 0.9 can be recommended.

RNAdeNoise performs best, giving 47 more genes compared to the raw data and 91 more compared to HTSFilter (29 and 21 genes by DESeq2 respectively). It is interesting to note that common approaches based on thresholds for minimal mRNA counts result in significantly fewer DEGs compared to the raw data. Classification of DEGs using DAVID [22] functional classification system shows an increase in genes with functional annotation after cleaning with RNAdeNoise, which exceeds the respective numbers by other filters (Table 1). We selected most populated functional classes “regulation of biological process” (GO:0050789), keyword “transcription regulation” (KW-0805) and “molecular function regulator” (GO:0098772) as examples. In most categories our method outperforms other methods in terms of number of identified functional genes. An interesting behavior shows a group-based filter “½ samples > 3” – the number of genes with function “regulation of biological process” is surprisingly high compared to other filters, but only in combination with the EdgeR program. In all other cases the performance of the filter is usual, even though it is better than that of the per-sample filters.

The distribution of DEGs according to the level of total mRNA shows the differential impact of pre-filtering on detected DEGs (Additional file 1: Table S2). Our method leads to more identified genes with a moderate level of transcription (+ 47 genes out of 844) compared to the raw data. The application of other filters strongly reduces the number of low-transcription genes, and has a minor effect on moderately expressed genes, with the FPKM filter showing the strongest reduction. The detection of highly transcribed DEGs is indifferent to any type of filtering.

**Table 1:** Data cleaning increases the number of genes with functional annotation

Cleaning method	#genes after cleaning	#DEGs total*	#DEGs with			
			Any annotated function	Regulation of biological process	Transcription regulation	Molecular function regulator
RNAdeNoise	25,356	2439	2144	338	92	40
Raw data	37,336	2392	2086	326	87	38
HTSFilter	22,907	2348	2056	319	83	38
counts > 3	26,089	2309	2025	320	84	38
counts > 5	25,215	2287	2005	318	85	36
counts > 10	23,973	2128	1898	310	85	38
FPKM > 0.3	23,237	1930	1770	304	87	34
½samples > 3	24,173	2363	2063	360	84	38

Differentially translated genes were identified using EdgeR and annotated using DAVID classification system. Presented are the three most populated categories related to regulation. Results using DESeq2 are similar and can be found in  (Additional file 1: Table S3)

### Application of data cleaning to other datasets

To show a wide applicability of our methodology, three recently published RNA-seq datasets have been chosen, covering two organisms – *Arabidopsis thaliana* and mouse, and two most common sequencing technologies – Illumina and BGI.

The first dataset is the result of work on the comparison of gene expression between Alport mice and wild mice [23]. Data (SRR11206238 and SRR11206239) were converted into mRNA counts using *STAR* and *FeatureCounts* on Galaxy [24]. The distributions of the counts reveal a regular shape (Additional file 1: Fig. S2), and can be characterized by the mode at around 1750 counts for the sample SRR11206239 and 2600 for SRR11206238, which indicates a very high sequencing depth. Another distinguishing feature is a strong fluctuation of the near-zero counts and a generally high level of low reads. Nevertheless, data modeling and cleaning make the distributions close to negative binomial, as would be expected (Fig. S2). DEGs were detected using EdgeR after application of different filters and annotated by DAVID as above. The criterion for DEGs was relaxed to |log2(FoldChange)|>1 to match the number of genes reported by the authors in Tables 1, 2, 3 and 4 [23] when using raw data (Table 2, gene lists are at the project home page on GitHub).

**Table 2:** Cleaning data with high sequencing depth [23]

Cleaning method	#DEGs*(DEGs30^&^)	#DEGs/*p*-value				
		Single-organism process	Biological regulation	Regulation of biological process	Multicellular organism process	Response to stimulus
Author’s^$^	130 (29)	27/9.3e-3	24/2.3e-2	21/1.6e-1	21/1.9e-3	21/8.2e-3
Raw data	135 (96)	65/3.9e-2	52/3.4e-1	47/6.0e-1	7/9.4e-1	40/3.3e-1
RNAdeNoise	336 (328)	193/1.7e-4	164/5.4e-3	155/1.6e-2	33/3.1e-1	134/6.3e-4
HTSFilter	27 (27)	20/1.2e-1	14/7.4e-1	10/9.8e-1	13/1.8e-1	10/7.9e-1
counts > 3	184 (96)	65/3.9e-2	52/3.4e-1	47/6.0e-1	38/2.1e-1	40/3.3e-1
counts > 5	250 (98)	67/3.1e-2	54/2.8e-1	49/5.3e-1	40/1.5e-1	42/2.5e-1
counts > 10	572 (128)	76/5.1e-2	63/2.2e-1	58/4.0e-1	11/7.4e-1	52/6.8e-2
FPKM > 0.3	550 (166)	108/2.7e-2	91/1.1e-1	85/2.2e-1	15/8.0e-1	69/1.8e-1

This dataset illustrates a “step” phenomenon appearing after application of threshold–based filters, when one of the values under the threshold is zeroed. As a result, a program for DEGs detection preferentially finds genes with very low counts (in brackets genes with counts ≥ 30). RNAdeNoise does not introduce such a bias and shows an increase in number and statistical significance of functional DEGs (distributions of counts in DEGs is shown in Additional file 1: Fig. S3)

^$^Genes from Tables 1, 2, 3 and 4 [23]

*Criteria for DEGs: |log2(FoldChange)|>1.0, *p*-value < 0.0001

^&^DEGs with counts ≥ 30 at least in one sample

As follows from the table, the application of has a significant impact on the number of detected differential genes. Most filters significantly increase the number of DEGs, and only two – “HTSFilter” and “Σ > 20” decrease. A detailed view revealed many examples of DEGs with raw counts slightly above/below the threshold. This may indicate that these genes had been recognized as DEGs only when one of the counts was zeroed after cleaning, and the increased difference was recognized as significant. Therefore, we have complemented the DEGs criteria by requiring that raw counts exceed 30 (twice the noise level) in at least one sample. This drastically reduced the number of detected DEGs, for example, down to 128 DEGs (to 22.4%) by “counts > 10” and to 166 (33.2%) by “FPKM > 0.3” filters (Table 2). In contrast, RNAdeNoise shows a very small portion of low-count DEGs within a strongly increased number of detected DEGs. The distribution of genes in the five most populated functional classes shows, that RNAdeNoise not only increases the number of detected genes, but also the significance of GO overrepresentation.

The demonstrated effect of the preferred detection of low-count DEGs is so pronounced in this dataset, due to the large number of low-count genes, which is in turn a result of the high sequencing depth (Additional file 1: Fig. S2). Interestingly, the method used by the authors reveals an even greater bias towards low-count genes. 101 out of 130 DEGs have counts in both samples under 30, and 89 genes have both counts under 16 – the limit defined here to separate random reads (gene list in Additional file 1: Table S4). Such DEGs are obvious statistical artifacts, which usually occur when near-zero ratios are computed. Assuming the high sequencing depth (mode above 1700 reads) such genes can be classified as not expressed at all, rather than differentially expressed. The distributions of the raw counts in detected DEGs show that RNAdeNoise does not shift the peak of the distribution relative to that of raw data (Additional file 1: Fig. S3). In contrast, threshold–based filters and the method used by the authors introduce a significant bias towards low–count DEGs. This is another fundamental difference between threshold–based filters and RNAdeNoise, which suppresses noise equally from all genes.

A second example comes from an investigation of gene response to Cycloastragenol, the molecule that stimulates telomerase activity, cell proliferation and is supposed to help plants overcome different environmental stresses [25]. Using BGISEQ-500 platform, two samples were sequenced – treated with cycloastragenol and control (NCBI SRA PRJNA665188). The distributions of counts reveal a typical curve with a very high portion of reads close to zero and the mode at about 1200 (Additional file 1: Fig. S4). Modeling this data using RNAdeNoise gives values 15 and 19, which should be subtracted from reads from samples 1A and 1B, respectively (samples names according to the original publication). This completely removes the exponential part and fits the distribution to the one expected theoretically, i.e., to the negative binomial.

In identifying DEGs the authors refer to the theoretical approach published in 1997 [26] for which no software implementation exists. So we used EdgeR with the same criterion as the authors – |log_2_(FoldChange)|≥ 1 and slightly increased statistical significance (*p*-value ≤ 0.002) to match the number of DEGs published. Annotation was done by DAVID and the results compared to the published results (Table 3, gene lists are at the project home page on GitHub).

**Table 3:** Results of the cleaning of expression data from BGISEQ-500 platform [25]

Cleaning method	#DEGs* (*p*-value^#^)	DEGs functional classification			
		Cellular process	Metabolic process	Response to stimulus	Regulation of biological process
Author’s^$^	1045	466	448	297	219
Raw data	1047 (6.77e-17)	501	458	314	211
RNAdeNoise	1215 (3.86e-18)	559	502	347	235
HTSFilter	860 (8.37e-17)	416	372	278	179
counts > 3	995 (5.58e-17)	477	434	301	201
counts > 5	988 (3.52e-17)	477	432	302	199
counts > 10	940 (4.97e-17)	450	409	293	183
FPKM > 0.3	790 (6.77e-17)	384	348	258	165

DEGs were detected by EdgeR and classified using DAVID. Filtering with RNAdeNoise increases the significance and adds 16% more DEGs and up to 20% DEGs in several functional classes compared to the original results

*Criteria for DEGs: |log2(FoldChange)|>1.0, *p*-value < 0.002

^#^Average *p*-value of top 100 genes

^$^First four functional classes from Fig. 3 [25] were taken for comparison

The results in the table show, that after filtering with RNAdeNoise the DEGs become more significant *p*-values compared to the raw data and other filters, which translates into an 16% increase in the number of DEGs (1215 vs. 1047), with a corresponding increase in all functional groups. None of the other filters were able to increase the number of detected genes. Generally, the improvement is comparable to that shown in Table 1, except that HTSFilter performed poorly on this data. Of note, the use of EdgeR compared to the author’s method [26] has itself increased the number of genes in three functional classes. Distribution according to the expression level demonstrates that the genes most affected by cleaning, have expression levels below approx. 1.5 log_2_CPMs ( Additional file 1: Fig. S5), which corresponds to 195 raw counts or 1/6 of the mode.

The third dataset used here as an example originates from the study of a circadian clock in *Arabidopsis thaliana* [27]. The dataset represents a special interest, because of extensive use of sample multiplexing in sequencing, which may or may not influence the shape of the exponential part, and therefore, the applicability of the method. The experiment consisted in measuring total mRNA and mRNA isolated using Translating Ribosome Affinity Purification (TRAPed mRNA) using Illumina sequencing technology and includes altogether 148 samples (NCBI GEO id GSE158444). We selected four datasets covering all types of mRNA source, i.e., total and TRAPed mRNAs from control and treated plants, to show the applicability of our technology (samples TOT.T0.C1, TOT.T0.H1, TR.T0.C1, TR.T0.H1), and six datasets (control vs. treated) to show the added value of cleaning (samples TOT.T0.C1, TOT.T0.C2, TOT.T0.C3 vs. TOT.T0.H1, TOT.T0.H2, TOT.T0.H3).

The distribution of counts reveals a typical shape, with a mode at about 100 counts for three samples and about 300 counts for one sample (Additional file 1: Fig. S6). Application of RNAdeNoise yields in bell-shaped distributions, which proves the applicability of the suggested approach.

One of the distinctive characteristics of this dataset is the mode of about 100 counts, which is seven times less compared to our work and twelve times compared to [25]. So we can estimate that up to eight samples were multiplexed in a single sequencing run. Data modeling gave a value of 12 for samples with a mode of 100 and 11 for the sample with a mode of 300, which was obviously less multiplexed. This can be interpreted as the noise level remains the same, while the “real counts” are proportionally reduced when the samples are multiplexed. In practice, this is important in determining how many samples can be multiplexed without compromising the ability to detect differential genes. For example, in this dataset 33% of genes with the lowest expression have mRNA counts below 30, of which up to 12 counts may be of a random nature. In such a case, it would be impossible to analyze the weakly expressed genes. To detect rare mRNAs, a much higher sequencing depth is required, and data modeling can be used to estimate the level of noise and the number of samples that can still be multiplexed.

To show the added value of RNAdeNoise on such multiplexed datasets, it was applied together with other filters to six samples, followed by DESeq2 as in the original publication (Table 4, genes lists are at the project home page on GitHub). Filtering used by the authors was also applied – the sum of all counts in control and treated samples (each in three repetitions) for a gene should be above 20, otherwise the gene is removed [27]. The comparative results show that the number of differential genes detected after cleaning with RNAdeNoise increased significantly (+ 20%). The average *p*-value over the top 1000 genes showed higher significance after cleaning. Similarly, the expression ratios are also increased – the average log2(FoldChange) for the top 1000 upregulated genes equals 3.402 after RNAdeNoise, 3.060 for raw data and 3.310 for count > 10 filter. Other filtering methods including author’s filtering (column “Σ > 20”) show weaker *p-*values and fewer detected genes. In this example, we do not functionally classify genes, since the original work aimed at finding circadian genes in a quite complex scheme with four types of samples over eight consecutive time points. The search for functional genes at a single time point in this case would have no biological relevance to the experiment.

**Table 4:** Cleaning the data from multiplexed samples [27]

Cleaning method	Σ > 20*	Raw	RNAdeNoise	HTSFilter	counts > 3	counts > 5	counts > 10	FPKM > 0.3
#DEGs^$^(*p*-value^#^)	2489 (5.4e-21)	2425 (1.1e-20)	2909 (8.3e-22)	2491 (5.8e-21)	2446 (1.0e-20)	2470 (3.1e-20)	2394 (6.8e-21)	2615 (4.7e-21)

Raw data was filtered using the respective filters, followed by DESeq2 for detection of DEGs. RNAdeNoise adds 17% more DEGs compared to the author’s results

*Filter used by the authors – sum of reads in all samples is above 20

^$^Criteria for DEGs: |log2(FoldChange)|>1.5, *p-*value < 0.0001

^#^Average *p*-value of top 1000 genes

Taking together, the above examples demonstrate: *i)* data modeling and de-noising, increase statistical significance of detected DEGs, which is transferred to the increase in the overall number of genes and genes with annotated functionality; *ii)* the effect of the data cleaning is more pronounced on low expression genes and in single replicate experiments; *iii*) the method is applicable to common sequencing technologies like Illumina and BGI, all organisms and robust against sample multiplexing; and finally *iv)* the method automatically adjusts its parameters to the data, eliminating subjectivity in selecting appropriate thresholds for minimal counts.

## Discussion

Advances in DNA sequencing have revolutionized genetic studies with a variety of sequencing technologies, which are used to investigate gene expression through mRNA quantification. Bioinformatic processing of such data is actively developing in many ways, including problem-specific tasks like data-normalization and low reads filtering [7]. A step of data filtering (or cleaning) can be included in a typical RNA-seq pipeline, either directly, in the form of a minimal required counts, or implicitly, when a program for DEGs detection already incorporates the cleaning [14].

The conceptual difference of the presented method consists in the assumption that all reads receive some level of noise, independent from the actual level (high or low) of mRNA. Therefore, in order to eliminate noise a certain value should be subtracted from all mRNAs. The method has another very important point – it introduces fewer artifacts, compared, for example, to fixed thresholds. As seen in the mouse dataset (Table 2), the use of fixed thresholds introduces a “step” in pairs of values slightly below/above the threshold, which can be further recognized as a differential expression. The increase in sequencing depth, which is intended to enhance resolution at low-level transcripts, in practice leads to a dramatic increase in low-count DEGs, which are obvious statistical artifacts introduced either by thresholds-based filters or by DEG detection program itself. This problem may not be so pronounced if the number of low-count genes is small, but still requires attention.

Cleaning of RNA-seq data has a predominant effect on recognition of DEGs with low to moderate transcription, which does not underestimate the significance of these genes and the method. For example, regulatory genes, encoding transcription factors and other regulatory proteins, including so-called master regulator genes, are typically reveal low to moderate transcription [28, 29], but have a great influence on the organism development and are the key elements in response to external and internal signals [30]. Taking that such genes are actively transcriptionally and translationally regulated, it may become difficult to detect deferential expression of these genes in the presence of (i) structural genes with high absolute expression statistically masking genes with lower expression; (ii) sequencing noise, reducing the contrast between expression levels. The latter can be improved through the data cleaning, based on explicit mathematical modeling excluding subjectivity. As we demonstrated on our data and on three other datasets, the cleaning using RNAdeNoise increases statistical significance, overall number of detected differential genes, genes with functional annotation and improves overrepresentation statistics in functional classes.

The current limitations of the method include, first and foremost, the need for manual control of the shape of the distributions. The prerequisite is a two-peak shape, which is interpreted as consisting of real and random parts. But in practice it is not always possible to reach sufficient sequencing depth, which can be seen as a reduced or missing second peak. For example, because of the in vivo collection of specific immune cells of mouse thymus, only a very few number of cells can be isolated for sequencing [31]. Thus, the required sequencing depth cannot be achieved and the distribution has only one peak (Additional file 1: Fig. S7). Another reason could be the sequencing technology. Distribution of mRNA counts of polysomal and monosomal tomato RNA fractions, sequenced on the MinION device (nanoporetech.com) similarly shows only an exponent-shaped distribution (Fig. S7), although many genes have counts far above the commonly used thresholds of 3 to 10. In both above cases it would be wrong to interpret the data to be purely random, but so that it is not possible to separate noise and real reads using statistics. Similarly, RNAdeNoise should not be used if the exponential part is missing, for example, if the data has already been cleaned. Iterative use may result in incorrect exponential model fitting and data corruption. Automatic detection of correctness of the input data will be the primary focus for future program development.

## Conclusion

Here we have presented a program RNAdeNoise for cleaning RNA-seq data, which improves the detection of differentially expressed genes and specifically genes with a low to moderate absolute level of transcription. Based on a data modeling approach, parameters of randomly distributed mRNAs are identified and the reads, most probably originating from a technical noise, are removed. We demonstrate that the elimination of this random component results in detection of more genes with more significant *p*-values compared to the use of common filters.

Another important characteristics of the method is its adaptation to data – the noise level is independently measured for each dataset and once no noise is detected, the data is left unaltered. This makes integration into existing analysis pipelines trivial and requires minimal user intervention. The method can also be applied to any dataset that comprises exponential and bell-shaped parts, probably with minor modifications to the provided program code. A practical advantage of RNAdeNoise is that it has only one tunable parameter – the filtering strength, which can be left at its default value of 0.9 in most cases. Examples of usage can be found in the supplementary files and at GitHub.

## Supplementary information 

**Additional file 1.** Supplementary figures, tables and gene lists.

**Additional file 2.** R program and examples. The program code of RNAdeNoise in R language, examples of the use.

## Data Availability

All data generated and analysed during this study are included in this published article and its supplementary information file, and also available at https://github.com/Deyneko/RNAdeNoise.

## Abbreviations

DEGs: Differentially expressed genes
FPKM/RPKM: Frequency/Reads Per Kilobase per Million reads
TMM: Normalization procedure in EdgeR
log2(FoldChange): Logarithm with base 2 of the ratio of two count values
counts>3/5/10: Filter genes with count values below or equal 3/5/10
Σ>20: Filter genes with the sum of counts in all samples equal or below 20
½samples>3/5/10: Filter genes if in half of the samples count values are below or equal 3/5/10
