# Supplementary material for: Modeling and cleaning RNA-seq data significantly improve detection of differentially expressed genes
Source: BMC Bioinformatics. 2022 Nov 16;23:488. doi: 10.1186/s12859-022-05023-z (PMC9670425; doi:10.1186/s12859-022-05023-z)
Supplement: Supplementary file 1 — Additional file 1. Supplementary figures, tables and gene lists. [file 12859_2022_5023_MOESM1_ESM.pdf]

Supplementary materials for the article: “Modeling and cleaning of RNA-seq data significantly improve detection of differentially expressed genes.”

Igor V. Deyneko[1*], Orkhan N. Mustafaev[2], Alexander A. Tyurin[1], Ksenya V. Zhukova[1], Alexander Varzari[3] and Irina V. Goldenkova-Pavlova[1*]

[1]K.A. Timiryazev Institute of Plant Physiology RAS, IPP RAS, Moscow, Russia

[2]Genetic Resources Institute, Azerbaijan National Academy of Sciences, Baku, Azerbaijan

[3]Laboratory of Human Genetics, Chiril Draganiuc Institute of Phthisiopneumology, Kishinev, Republic of Moldova.

*Corresponding authors

E-mail: igor.deyneko@inbox.ru (IVD), irengold58@gmail.com (IVG-P)

Figure S1. Distribution of p-values. Histograms of p-values of DEGs after different filters. Histograms in the background (grey) represent p-values of raw data, in foreground – filtered data. **A**. Identification of differential genes using EdgeR. **B**. Identification of differential genes using DESeq2. Of note, DESeq2 automatically filters genes according to internal criteria, so most of genes with p-value≈1 are removed.

A

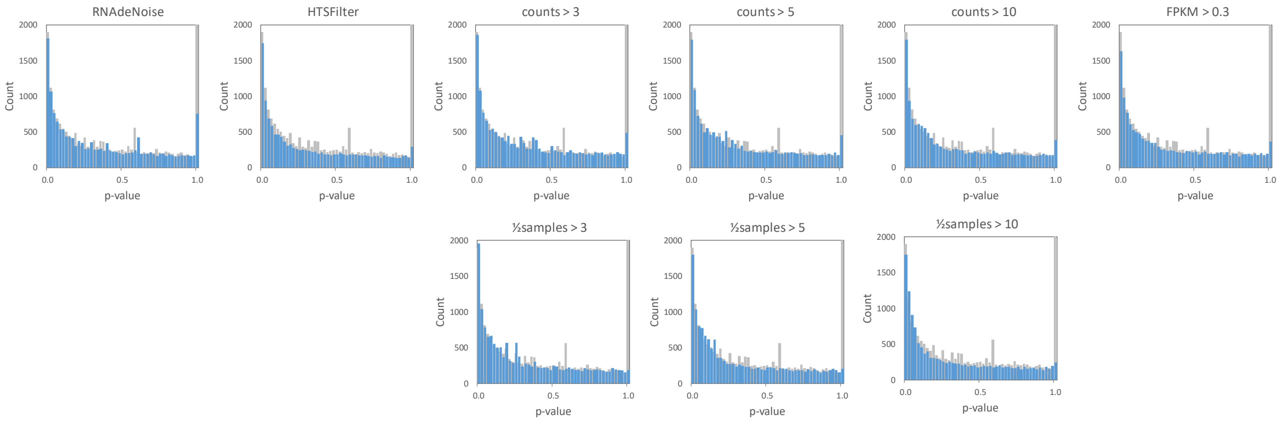

B

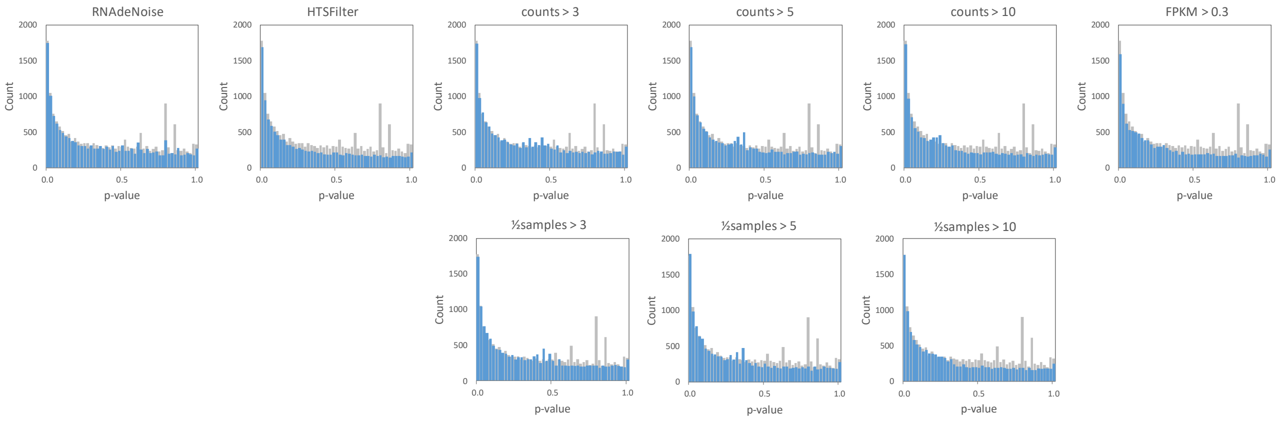

Table S1. 24 genes uniquely identified with RNAdeNoise. Differentially translated genes were identified using EdgeR (polysomal vs. monosomal fractions) after filtering with RNAdeNoise, but missed by all other filters.

Geneid	Map_STAR.1_1.1.sorted.bam	Map_STAR.2_2.1.sorted.bam	Map_STAR.3_3.1.sorted.bam	Map_STAR.4_1.2.sorted.bam	Map_STAR.5_2.2.sorted.bam	Map_STAR.6_3.2.sorted.bam	logFC	logCPM	PValue	FDR
AT1G03550	322	430	225	101	131	178	-1.51423	2.255674	4.76E-06	5.54E-05
AT1G05780	202	440	258	106	126	140	-1.51332	2.131639	1.61E-05	0.000161
AT1G06630	650	1251	1058	316	370	546	-1.50298	3.858035	2.72E-08	5.57E-07
AT1G19340	219	288	298	110	119	102	-1.5016	1.972815	2.73E-06	3.39E-05
AT1G80240	74	100	108	276	321	294	1.51988	1.933405	2.03E-06	2.62E-05
AT2G16650	769	520	731	196	289	356	-1.50615	3.310838	2.16E-08	4.53E-07
AT2G22840	654	892	931	306	241	493	-1.50501	3.601411	4.51E-09	1.12E-07
AT2G40030	103	118	93	296	277	446	1.510168	2.091336	3.68E-07	5.71E-06
AT3G01430	622	652	510	252	272	192	-1.50638	3.131799	1.75E-07	2.95E-06
AT3G15400	180	202	174	59	74	102	-1.52339	1.429428	6.62E-06	7.40E-05
AT3G49645	370	484	526	175	159	241	-1.50493	2.754183	1.46E-08	3.18E-07
AT3G51075	191	141	187	55	68	97	-1.52594	1.328661	1.86E-05	0.000183
AT3G58130	336	412	391	140	109	230	-1.51933	2.473306	1.80E-07	3.03E-06
AT4G02485	249	376	349	95	135	176	-1.51842	2.244631	9.47E-07	1.32E-05
AT4G03115	235	270	273	101	99	121	-1.52335	1.918631	2.64E-07	4.24E-06
AT4G03635	463	479	524	210	112	295	-1.51291	2.84191	4.53E-07	6.86E-06
AT4G31248	670	523	693	189	274	318	-1.50802	3.209898	6.31E-09	1.51E-07
AT4G31250	670	523	693	189	274	318	-1.50802	3.209898	6.32E-09	1.51E-07
AT4G36050	421	620	545	180	162	327	-1.51039	2.954894	5.38E-08	1.03E-06
AT4G38660	117	192	108	447	468	399	1.50667	2.508807	3.55E-06	4.30E-05
AT5G02850	237	126	134	492	487	630	1.501071	2.771102	1.09E-06	1.51E-05
AT5G18040	321	540	463	147	183	215	-1.51486	2.691345	1.30E-07	2.28E-06
AT5G41850	262	212	250	97	74	135	-1.51446	1.817936	2.03E-06	2.62E-05
AT5G44150	383	445	416	155	158	205	-1.50243	2.606919	9.29E-09	2.14E-07

Table S2. Number of detected DEGs after different cleaning procedures. Differentially expressed genes were identified using EdgeR and DESeq2 in polysomal vs. monosomal fractions, after applying different cleaning methods – the presented approach, raw data (without cleaning), HTSfilter, fixed threshold of 3, 5 and 10 counts, FPKM > 0.3 and half of all samples should be above a threshold (½samples>3,5,10). Results are sorted according to the number of DEGs identified by EdgeR. Additionally, DEGs are classified using the total mRNA fraction into the three categories: low transcribed (lowest 1/3 quantile, ≤268 counts), moderate (middle 1/3 quantile, 269...1305 counts) and highly transcribed (top 1/3 quantile, ≥1306 counts). RNAdeNoise increases the number of DEGs in moderately expressed group of genes.

Cleaning method	#genes after cleaning	EdgeR				DESeq2			
		#DEGs	#DEGs by total mRNA			#DEGs	#DEGs by total mRNA		
		total*	Low	Mid	High	total*	Low	Mid	High
RNAdeNoise	25356	2439<br>+47**	848<br>+3	844<br>+47	747<br>-3	2254<br>+29	666<br>-21	846<br>+47	742<br>+3
Raw data	37336	2392	845	797	750	2225	687	799	739
HTSFilter	22907	2348<br>-44	803<br>-42	801<br>+4	744<br>-6	2233<br>+8	693<br>+6	800<br>+1	740<br>+1
counts > 3	26089	2309<br>-83	766<br>-79	800<br>+3	743<br>-7	2084<br>-141	552<br>-135	797<br>-2	735<br>-4
counts > 5	25215	2287<br>-105	738<br>-107	807<br>+10	742<br>-8	2055<br>-170	530<br>-157	786<br>-13	739<br>0
counts > 10	23973	2128<br>-264	593<br>-252	798<br>+1	737<br>-13	2049<br>-176	548<br>-139	767<br>-32	734<br>-5
FPKM > 0.3	23237	1930<br>-462	472<br>-373	722<br>-75	736<br>-14	2009<br>-216	556<br>-131	722<br>-77	731<br>-8
½samples>3	24173	2363<br>-29	815<br>-30	801<br>+4	747<br>-3	2133<br>-92	597<br>-90	795<br>-4	741<br>+2
½samples>5	23549	2325<br>-67	780<br>-65	799<br>+2	746<br>-4	2098<br>-127	564<br>-123	793<br>-6	741<br>+2
½samples>10	22573	2238<br>-154	692<br>-153	805<br>+8	741<br>-9	2047<br>-178	518<br>-169	787<br>-12	742<br>+3

* Criteria for DEGs: |log2(FoldChange)|>1.5, p-value<0.0001.

** Increase or decrease compared to the Raw data.

Table S3. Functional classification of DEGs. Differentially translated genes were identified using EdgeR and DESeq2 (polysomal vs. monosomal fractions) and annotated using DAVID classification system. Three example categories related to gene regulation are presented – "regulation of biological process" (GO:0050789), keyword "transcription regulation" (KW-0805) and "molecular function regulator" (GO:0098772). Shown are absolute number of DEGs and relative number either to total number of DEGs or to total number of annotated DEGs. Relative numbers are comparable and are only slightly higher for "FPKM>0.3"and "count>10" filters, the filters which significantly reduce the absolute number of detected genes.

Cleaning method	EdgeR, #DEGs				DESeq2, #DEGs			
	any annotated function (of total DEGs)	regulation of biological process (of annotated DEGs)	Transcription regulation (of annotated DEGs)	molecular function regulator (of annotated DEGs)	any annotated function	regulation of biological process	Transcription regulation	molecular function regulator
RNAdeNoise	2144 (0.879)	338 (0.158)	92 (0.043)	40 (0.019)	2013 (0.893)	324 (0.161)	88 (0.044)	35 (0.017)
Raw data	2086 (0.872)	326 (0.156)	87 (0.042)	38 (0.018)	1977 (0.889)	314 (0.159)	82 (0.041)	35 (0.018)
HTSFilter	2056 (0.876)	319 (0.155)	83 (0.04)	38 (0.018)	1984 (0.888)	314 (0.158)	82 (0.041)	35 (0.018)
counts >3	2025 (0.877)	320 (0.158)	84 (0.041)	38 (0.019)	1874 (0.899)	304 (0.162)	79 (0.042)	35 (0.019)
counts > 5	2005 (0.877)	318 (0.159)	85 (0.042)	36 (0.018)	1847 (0.899)	305 (0.165)	78 (0.042)	34 (0.018)
counts > 10	1898 (0.892)	310 (0.163)	85 (0.045)	38 (0.02)	1841 (0.898)	298 (0.162)	79 (0.043)	37 (0.02)
FPKM>0.3	1770 (0.917)	304 (0.172)	87 (0.049)	34 (0.019)	1830 (0.911)	306 (0.167)	85 (0.046)	38 (0.021)
½samples>3	2063 (0.921)	360 (0.174)	84 (0.041)	38 (0.018)	1912 (0.934)	308 (0.162)	81 (0.042)	32 (0.017)

Figure S2. Distribution of mRNA counts in data from [Dufek, 2021]. Distributions of RNA-seq data – original data, cleaned and exponential estimation. Sample names according to original publication.

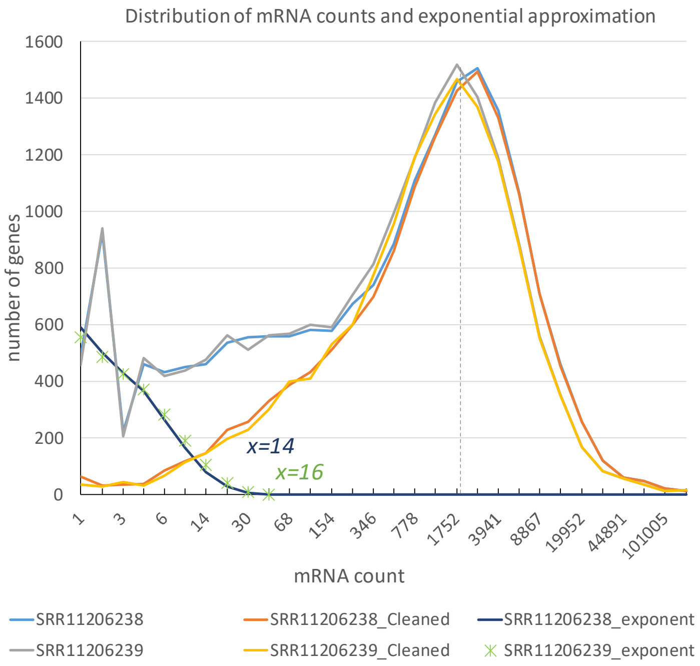

Figure S3. Distribution of raw mRNA counts in DEGs detected using different filters and by Ingenuity Pathway Analysis (IPA) software on data from [Dufek, 2021]. RNAdeNoise and raw data have a mode at around 50 counts. Threshold–based filters introduce bias into the data, so that the program for DEGs detection (EdgeR) preferentially finds DEGs with lower counts. Even stronger bias shows IPA used by the authors. Grey zone up to 16 counts indicates reads which, most probably originated from the random noise.

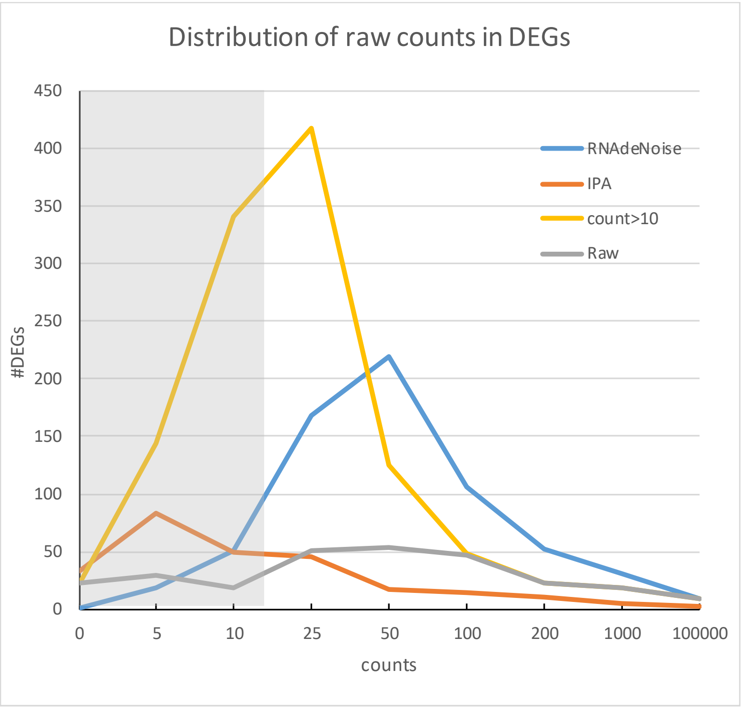

Figure S4. Distribution of mRNA counts in raw and cleaned data. RNA-seq data on *Arabidopsis thaliana* sequenced on BGISEQ-500 platform [Mhiri, et al., 2020]. Shown are raw mRNA reads, cleaned reads and the exponentially distributed part for samples 1A (treated with Cycloastragenol) and 1B (control). Modeling the data with RNAdeNoise gives that values of 15 and 19 should be subtracted from each mRNA raw count in samples 1A and 1B. Distribution of cleaned values (blue line) is similar to negative binomial, as theoretically expected.

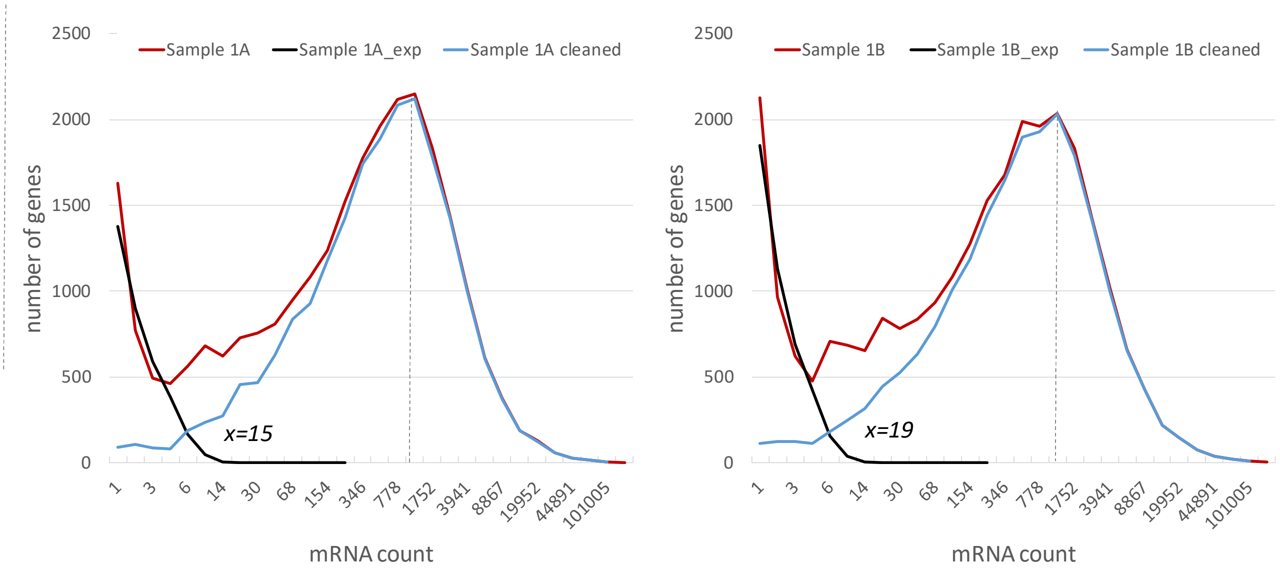

Figure S5. Distribution of mRNA counts in raw and cleaned data from Mhiri [26]. Distribution of DEGs according to absolute level of mRNA (logCPM).

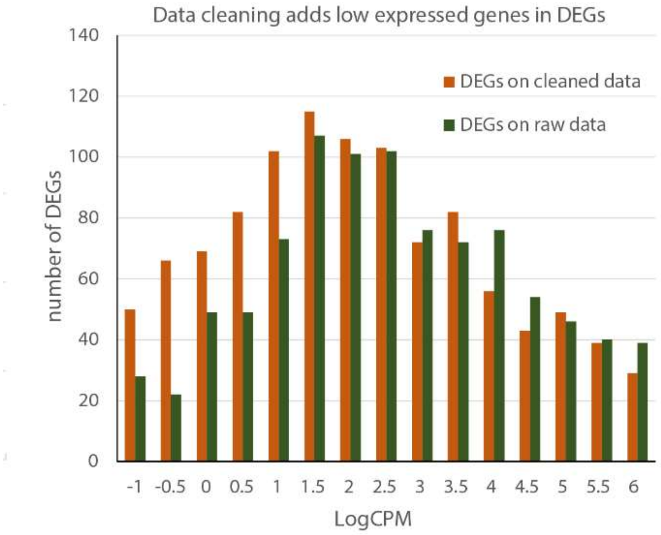

Figure S6. Distribution of mRNA counts in data from [Bonnot and Nagel, 2021]. Distributions of RNA-seq data – original, cleaned and exponential estimation. **A.** Overview of the four example datasets of original and cleaned data. **B.** Detailed figures for each dataset with original, cleaned and exponential estimation.

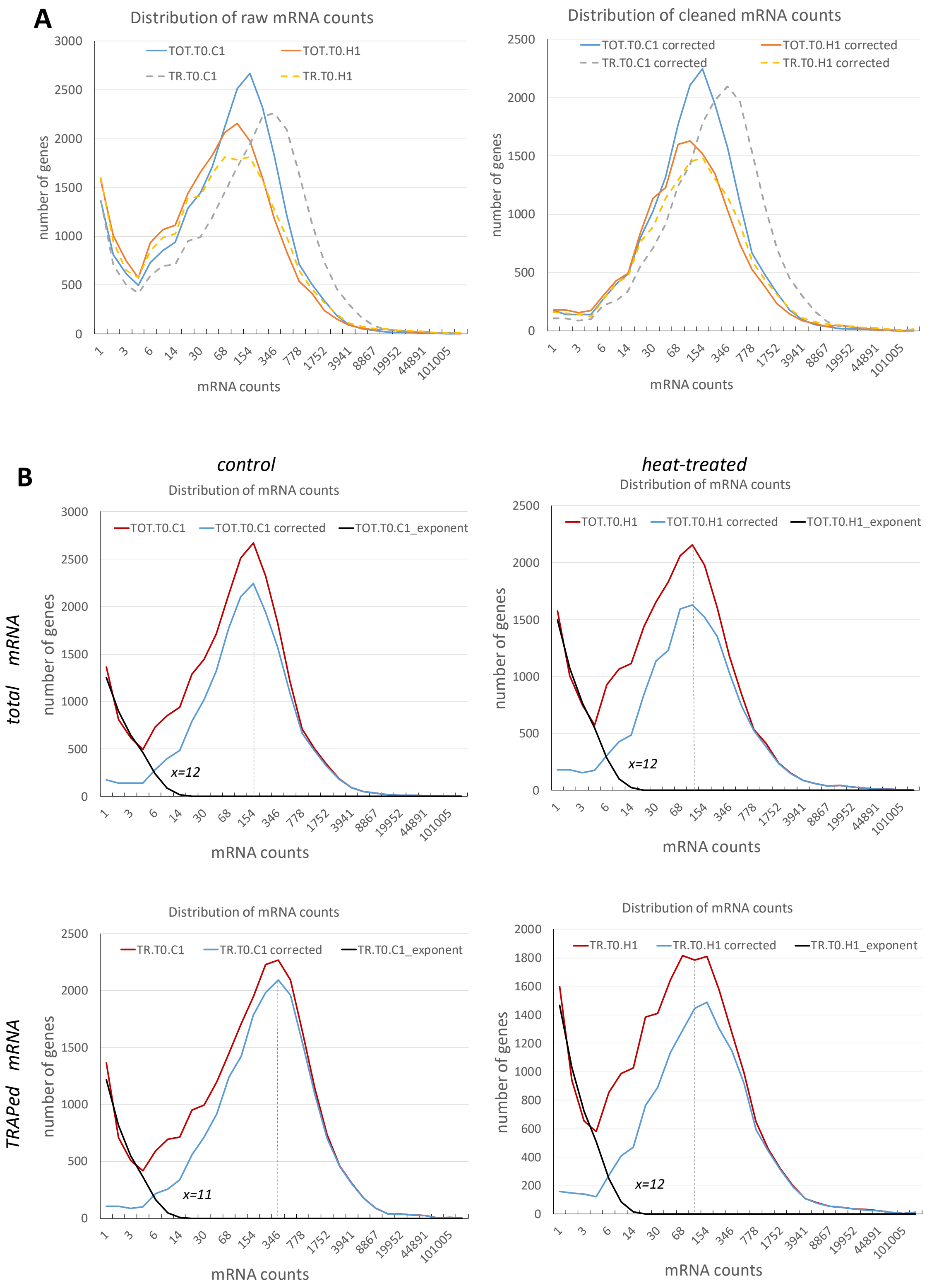

Figure S7. Distribution of mRNA counts not suitable for cleaning with RNAdeNoise.

A) Data from bisulfite sequencing on Illunina, distribution of methylated DNA fragments according to the number of reads per fragment [Yang et. al., 2015]. The distribution has only one peak, and therefore cleaning with RNAdeNoise will remove most of the reads. Here, sufficient sequencing depth cannot be reached due to the *in vivo* collection of TH17 cells from thymus and only a few cells were used for DNA extraction.

B) Distribution of mRNA counts from polysomal and monosomal RNA fractions from tomato, sequenced on MinION device (nanoporetech.com). Sequencing depth is far not enough to statistically separate noise and real reads (authors own data, not published).

A

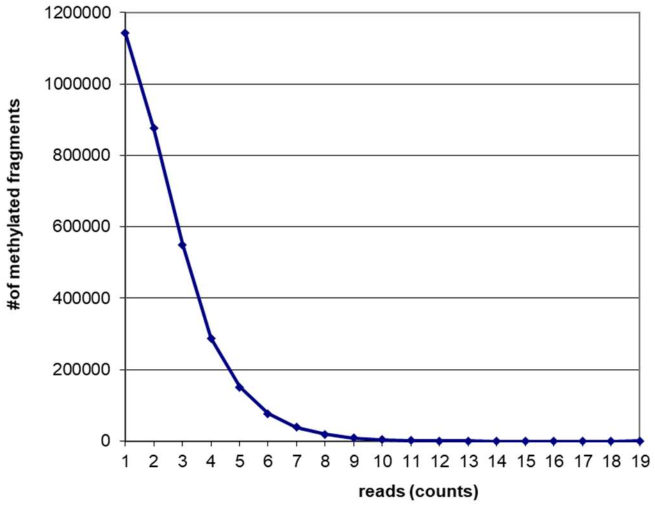

B

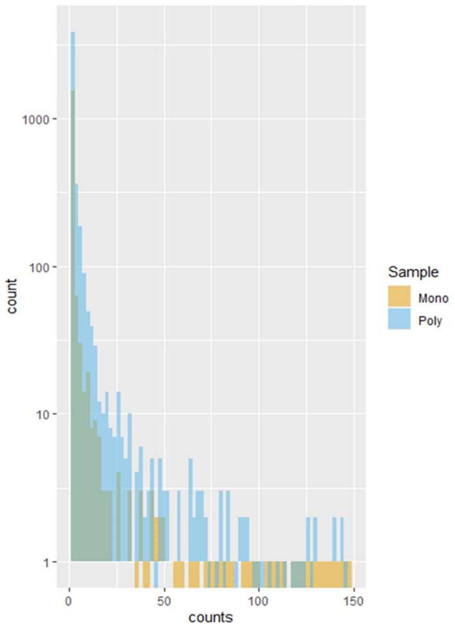

Table S4. Raw counts for genes from tables 1-4 [Dufek1 et al., 2021].

Gene stable ID	Gene name	NCBI gene ID	counts SRR11206238	counts SRR11206239
ENSMUSG00000006179	Prss16	54373	16	2
ENSMUSG00000027776	Il12a	16159	2	4
ENSMUSG00000048304	Slitrk3	386750	4	12
ENSMUSG00000035472	Slc25a21	217593	20	0
ENSMUSG00000048349	Pou4f1	18996	2	0
ENSMUSG00000031910	Has3	15118	13	2
ENSMUSG00000022651	Retnlg	245195	10	4
ENSMUSG00000021565	Slc6a19	74338	12	2
ENSMUSG00000030142	Clec4e	56619	10	4
ENSMUSG00000035910	Dcdc2a	195208	9	4
ENSMUSG00000039462	Col10a1	12813	7	5
ENSMUSG00000035805	Mlc1	170790	2	4
ENSMUSG00000029618	Ocm	18261	152	199
ENSMUSG00000032053	Pou2af1	18985	8	4
ENSMUSG00000037548	H2-DMb2	15000	18	1
ENSMUSG00000043448	Gjc2	118454	0	12
ENSMUSG00000005465	Il27ra	50931	52	33
ENSMUSG00000019935	Slc17a8	216227	6	8
ENSMUSG00000061974	Cldn24	1E+08	233	46
ENSMUSG00000022054	Nefm	18040	14	4
ENSMUSG00000031103	Elf4	56501	72	66
ENSMUSG00000020651	Slc26a4	23985	7673	2985
ENSMUSG00000073574	Grxcr2	332309	4	18
ENSMUSG00000002664	Pspn	19197	4	4
ENSMUSG00000030124	Lag3	16768	2	16
ENSMUSG00000028976	Slc2a5	56485	6	12
ENSMUSG00000037225	Fgf2	14173	3	2
ENSMUSG00000055561	Spink5	72432	773	224
ENSMUSG00000054191	Klf1	16596	16	0
ENSMUSG00000056073	Grik2	14806	11	16
ENSMUSG00000022762	Ncam2	17968	6	14
ENSMUSG00000029275	Gfi1	14581	3	11
ENSMUSG00000053519	Kcnip1	70357	4	1
ENSMUSG00000049649	Gpr3	14748	10	8
ENSMUSG00000024401	Tnf	21926	16	2
ENSMUSG00000024402	Lta	16992	6	0
ENSMUSG00000045534	Kcna5	16493	24	42
ENSMUSG00000047976	Kcna1	16485	14	6
ENSMUSG00000042686	Jph1	57339	60	29
ENSMUSG00000019893	Ros1	19886	32	2
ENSMUSG00000024673	Ms4a1	12482	7	0

ENSMUSG00000025318	Jph3	57340	0	2
ENSMUSG00000025221	Kcnip2	80906	0	0
ENSMUSG00000021614	Vcan	13003	4	11
ENSMUSG00000028072	Ntrk1	18211	2	0
ENSMUSG00000021070	Bdkrb2	12062	8	2
ENSMUSG00000079415	Cntf	12803	2	1
ENSMUSG00000041872	Il17f	257630	2	0
ENSMUSG00000028068	Iqgap3	404710	42	24
ENSMUSG00000078816	Prkcg	18752	35	11
ENSMUSG00000021732	Fgf10	14165	19	2
ENSMUSG00000056222	Spock1	20745	53	111
ENSMUSG00000004296	Il12b	16160	26	4
ENSMUSG00000051243	Islr2	320563	2	14
ENSMUSG00000038331	Satb2	212712	32	10
ENSMUSG00000068697	Myoz1	59011	2	8
ENSMUSG00000024749	Tmc1	13409	37	70
ENSMUSG00000026587	Astn1	11899	8	11
ENSMUSG00000046080	Clec9a	232414	0	7
ENSMUSG00000033576	Apol6	71939	2	0
ENSMUSG00000022683	Pla2g10	26565	2	8
ENSMUSG00000031162	Gata1	14460	2	0
ENSMUSG00000035681	Kcnc2	268345	4	0
ENSMUSG00000026770	Il2ra	16184	76	69
ENSMUSG00000005716	Pvalb	19293	6	0
ENSMUSG00000013974	Mcemp1	69189	6	4
ENSMUSG00000000632	Sez6	20370	4	2
ENSMUSG00000064065	Ipcef1	320495	15	0
ENSMUSG00000037405	Icam1	15894	187	101
ENSMUSG00000025582	Nptx1	18164	6	30
ENSMUSG00000028681	Ptch2	19207	6	6
ENSMUSG00000021803	Cdhr1	170677	0	6
ENSMUSG00000022208	Jph4	319984	2	4
ENSMUSG00000072596	Ear2	13587	0	2
ENSMUSG00000054641	Mmrn1	70945	2	4
ENSMUSG00000071424	Grid2	14804	42	8
ENSMUSG00000023391	Dlx2	13392	2	0
ENSMUSG00000001494	Sost	74499	0	4
ENSMUSG00000029075	Tnfrsf4	22163	2	7
ENSMUSG00000050473	Slc35d3	76157	8	3
ENSMUSG00000029254	Stap1	56792	5	11
ENSMUSG00000028039	Efna3	13638	7	3
ENSMUSG00000024245	Tmem178	68027	3	4
ENSMUSG00000040809	Chil3	12655	21	14
ENSMUSG00000054640	Slc8a1	20541	23	15
ENSMUSG00000042453	Reln	19699	2	4
ENSMUSG00000037446	Tulp1	22157	2	2
ENSMUSG00000032484	Ngp	18054	8	32
ENSMUSG00000062252	Lhfpl5	328789	31	80

ENSMUSG00000005763	Cd247	12503	7	4
ENSMUSG00000052212	Cd177	68891	6	6
ENSMUSG00000026117	Zap70	22637	6	10
ENSMUSG00000020704	Asic2	11418	0	2
ENSMUSG00000035373	Ccl7	20306	12	2
ENSMUSG00000009292	Trpm2	28240	49	21
ENSMUSG00000013921	Clip3	76686	112	147
ENSMUSG00000060961	Slc4a4	54403	70	120
ENSMUSG00000031845	Bco1	63857	10	3
ENSMUSG00000030827	Fgf21	56636	8	2
ENSMUSG00000020932	Gfap	14580	1	11
ENSMUSG00000020327	Fgf22	67112	6	2
ENSMUSG00000026418	Tnni1	21952	2	1
ENSMUSG00000095139	Pou3f2	18992	0	8
ENSMUSG00000040270	Bach2	12014	65	44
ENSMUSG00000058975	Kcnc1	16502	4	0
ENSMUSG00000004707	Ly9	17085	79	12
ENSMUSG00000029334	Prkg2	19092	2	0
ENSMUSG00000040026	Saa3	20210	0	4
ENSMUSG00000040592	Cd79b	15985	9	4
ENSMUSG00000028460	Sit1	54390	4	0
ENSMUSG00000079227	Ccr5	12774	91	42
ENSMUSG00000056978	Hamp2	66438	4	0
ENSMUSG00000028332	Hemgn	93966	0	2
ENSMUSG00000049723	Mmp12	17381	15	2
ENSMUSG00000028307	Aldob	230163	10	4
ENSMUSG00000020396	Nefh	380684	21	10
ENSMUSG00000078630	Tomt	791260	170	131
ENSMUSG00000034990	Otoa	246190	643	295
ENSMUSG00000036526	Card11	108723	7	0
ENSMUSG00000020798	Spns3	77577	54	14
ENSMUSG00000048583	Igf2	16002	11	8
ENSMUSG00000027120	Fshb	14308	4	8
ENSMUSG00000001506	Col1a1	12842	15	22
ENSMUSG00000048482	Bdnf	12064	4	8
ENSMUSG00000025515	Muc2	17831	2	14
ENSMUSG00000027077	Smtnl1	68678	6	16
ENSMUSG00000019122	Ccl9	20308	20	0
ENSMUSG00000000982	Ccl3	20302	4	20
ENSMUSG00000074934	Grem1	23892	10	0

Table S5. DEGs detected using RNAdeNoise filter Raw on data from [Dufek1 et al., 2021].

geneID (Authors)	logFC	logCPM	PValue	FDR	EnsID	raw counts SRR11206238	raw counts SRR11206239
14608	-9.10996	-0.32095	3.94E-16	1.07E-11	ENSMUSG00000031932	91	16
100039801	-2.52481	1.323507	9.44E-15	1.28E-10	ENSMUSG00000061974	233	46
73010	-8.90966	-0.50582	2.54E-14	1.72E-10	ENSMUSG00000044067	81	10
57394	-8.90966	-0.50582	2.54E-14	1.72E-10	ENSMUSG00000015401	81	14
17085	-8.86603	-0.54588	6.55E-14	3.56E-10	ENSMUSG00000004707	79	12
64176	-3.28293	0.308398	1.09E-13	4.96E-10	ENSMUSG00000053025	126	25
67412	8.767546	-0.76523	1.49E-13	5.80E-10	ENSMUSG00000038916	12	64
64293	-8.75081	-0.65134	4.48E-13	1.51E-09	ENSMUSG00000029123	74	15
170743	-5.55767	-0.5027	5.01E-13	1.51E-09	ENSMUSG00000044583	80	17
15891	-2.09616	1.633917	1.45E-12	3.93E-09	ENSMUSG00000029306	274	64
12395	-3.96435	-0.1779	3.11E-12	7.21E-09	ENSMUSG00000006586	95	20
15233	-8.65151	-0.74178	3.18E-12	7.21E-09	ENSMUSG00000022821	70	14
224129	-4.63123	-0.48003	4.21E-12	8.79E-09	ENSMUSG00000022840	80	18
504186	3.436205	-0.30109	7.35E-12	1.43E-08	ENSMUSG00000066279	21	77
272714	-8.59918	-0.78928	8.60E-12	1.56E-08	NA	68	16
140476	8.431382	-1.0841	2.80E-11	4.75E-08	ENSMUSG00000033498	7	54
14275	-3.11952	0.008896	3.49E-11	5.58E-08	ENSMUSG00000001827	103	24
22139	-1.64775	2.525295	6.95E-11	1.02E-07	ENSMUSG00000061808	470	131
74075	-2.71869	0.32798	7.14E-11	1.02E-07	ENSMUSG00000025480	123	29
72432	-1.52831	3.283347	8.07E-11	1.07E-07	ENSMUSG00000055561	773	224
270162	8.353611	-1.15697	8.24E-11	1.07E-07	ENSMUSG00000041986	4	52
214593	-8.23767	-1.11394	1.42E-10	1.76E-07	ENSMUSG00000068452	56	15
329152	-3.05328	-0.04737	1.49E-10	1.76E-07	ENSMUSG00000042807	99	24
15013	-8.39943	-0.96944	1.79E-10	2.03E-07	ENSMUSG00000091705	61	16
271970	-2.06213	1.122488	1.98E-10	2.16E-07	ENSMUSG00000046561	194	50
12919	-1.3046	7.937707	4.02E-10	3.62E-07	ENSMUSG00000021680	18454	5919
23972	-2.06951	0.999302	4.03E-10	3.62E-07	ENSMUSG00000024899	179	47
77577	-8.16752	-1.17619	4.27E-10	3.62E-07	ENSMUSG00000020798	54	14
70821	-8.16752	-1.17619	4.27E-10	3.62E-07	ENSMUSG00000029828	54	16
319998	-8.13112	-1.20839	4.27E-10	3.62E-07	ENSMUSG00000051703	53	16
67516	-8.13112	-1.20839	4.27E-10	3.62E-07	ENSMUSG00000046523	53	8
74016	-8.13112	-1.20839	4.27E-10	3.62E-07	ENSMUSG00000026873	53	14
791357	-8.33689	-1.02547	5.04E-10	4.15E-07	NA	59	4
20698	-1.91005	1.299459	6.21E-10	4.96E-07	ENSMUSG00000061878	214	58
71145	-8.09378	-1.24134	7.43E-10	5.46E-07	ENSMUSG00000022032	52	13
212517	-8.09378	-1.24134	7.43E-10	5.46E-07	ENSMUSG00000071550	52	9
245537	-8.09378	-1.24134	7.43E-10	5.46E-07	ENSMUSG00000031302	52	14
109889	-8.30457	-1.05434	8.47E-10	5.75E-07	ENSMUSG00000030380	58	10
12931	-8.30457	-1.05434	8.47E-10	5.75E-07	ENSMUSG00000007888	58	9
234673	-8.30457	-1.05434	8.47E-10	5.75E-07	ENSMUSG00000031886	58	10
216049	8.138594	-1.35646	1.30E-09	8.20E-07	ENSMUSG00000037855	11	47
23794	-8.05545	-1.27509	1.30E-09	8.20E-07	ENSMUSG00000022894	51	12
665180	-8.05545	-1.27509	1.30E-09	8.20E-07	ENSMUSG00000079598	51	2

636791	-3.83515	-0.6634	1.49E-09	9.22E-07	ENSMUSG00000094002	70	19
100038696	-4.28809	-0.78228	1.70E-09	1.03E-06	NA	66	18
71603	-8.01607	-1.30967	2.27E-09	1.34E-06	NA	50	10
14089	-1.64905	1.779608	3.31E-09	1.91E-06	ENSMUSG00000000392	284	84
212032	-7.97559	-1.34513	3.99E-09	2.22E-06	ENSMUSG00000025877	49	11
319405	-7.97559	-1.34513	3.99E-09	2.22E-06	ENSMUSG00000097466	49	10
12308	-4.23166	-0.83131	4.38E-09	2.38E-06	ENSMUSG00000003657	64	18
18191	-3.45844	-0.6165	4.53E-09	2.42E-06	ENSMUSG00000066392	71	20
269109	-1.86154	1.12165	4.68E-09	2.45E-06	ENSMUSG00000036815	189	54
19739	-7.93394	-1.38151	7.04E-09	3.24E-06	ENSMUSG00000020599	48	12
50720	-7.93394	-1.38151	7.04E-09	3.24E-06	ENSMUSG00000048279	48	10
14805	-7.93394	-1.38151	7.04E-09	3.24E-06	ENSMUSG00000022935	48	16
110751	-7.93394	-1.38151	7.04E-09	3.24E-06	ENSMUSG00000027318	48	13
78004	-7.93394	-1.38151	7.04E-09	3.24E-06	ENSMUSG00000045725	48	14
232975	-7.93394	-1.38151	7.04E-09	3.24E-06	ENSMUSG00000040907	48	14
12768	-7.93394	-1.38151	7.04E-09	3.24E-06	ENSMUSG00000025804	48	8
72668	-2.29724	0.266769	1.14E-08	5.15E-06	ENSMUSG00000054074	114	32
94227	-7.89105	-1.41886	1.25E-08	5.46E-06	ENSMUSG00000067780	47	15
11634	-7.89105	-1.41886	1.25E-08	5.46E-06	ENSMUSG00000000731	47	12
574418	-2.94281	-0.31818	1.29E-08	5.55E-06	ENSMUSG00000046110	83	23
100503338	2.998248	-0.67917	1.69E-08	7.19E-06	NA	21	61
640524	3.541323	-0.96395	1.84E-08	7.69E-06	ENSMUSG00000074899	18	54
56839	-4.90733	-1.07991	2.11E-08	8.47E-06	ENSMUSG00000067242	56	17
75563	-7.84685	-1.45724	2.21E-08	8.47E-06	ENSMUSG00000042707	46	11
75369	-7.84685	-1.45724	2.21E-08	8.47E-06	ENSMUSG00000105773	46	5
11690	-7.84685	-1.45724	2.21E-08	8.47E-06	ENSMUSG00000060063	46	14
381917	-7.84685	-1.45724	2.21E-08	8.47E-06	ENSMUSG00000052273	46	16
213438	-7.84685	-1.45724	2.21E-08	8.47E-06	ENSMUSG00000054293	46	8
269959	1.996646	0.526124	2.56E-08	9.66E-06	ENSMUSG00000070469	44	111
229933	-1.72291	1.221476	2.60E-08	9.66E-06	ENSMUSG00000036960	198	60
22029	-3.35373	-0.70502	2.63E-08	9.66E-06	ENSMUSG00000026875	67	20
329252	-4.87268	-1.10999	3.50E-08	1.27E-05	ENSMUSG00000042793	55	17
57746	7.885821	-1.58671	3.94E-08	1.36E-05	ENSMUSG00000033644	13	42
239845	7.885821	-1.58671	3.94E-08	1.36E-05	ENSMUSG00000046961	12	42
16819	-7.80124	-1.49671	3.94E-08	1.36E-05	ENSMUSG00000026822	45	13
102680	-7.80124	-1.49671	3.94E-08	1.36E-05	ENSMUSG00000036814	45	6
320100	-3.32633	-0.72806	4.09E-08	1.39E-05	ENSMUSG00000008318	66	20
14063	-2.0166	0.482189	5.64E-08	1.89E-05	ENSMUSG00000021678	127	38
80979	1.87484	0.566895	7.00E-08	2.28E-05	ENSMUSG00000029015	47	112
105975	-7.75415	-1.53732	7.04E-08	2.28E-05	ENSMUSG00000097209	44	8
67666	-7.75415	-1.53732	7.04E-08	2.28E-05	ENSMUSG00000030606	44	12
328789	2.241928	-0.07867	7.31E-08	2.34E-05	ENSMUSG00000062252	31	80
18973	3.229159	-0.93553	9.20E-08	2.91E-05	ENSMUSG00000007080	19	54
319776	-1.36773	2.294565	9.88E-08	3.07E-05	ENSMUSG00000048108	383	129
212190	-3.2699	-0.77529	9.94E-08	3.07E-05	ENSMUSG00000043621	64	20
68144	2.776792	-0.68268	1.01E-07	3.10E-05	NA	22	60
83395	7.770852	-1.68973	1.26E-07	3.47E-05	ENSMUSG00000038560	12	40
216166	-7.70547	-1.57915	1.26E-07	3.47E-05	ENSMUSG00000035486	43	12

380977	-7.70547	-1.57915	1.26E-07	3.47E-05	ENSMUSG00000097915	43	14
57276	-7.70547	-1.57915	1.26E-07	3.47E-05	ENSMUSG00000001943	43	12
12442	-7.70547	-1.57915	1.26E-07	3.47E-05	ENSMUSG00000032218	43	14
69352	-7.6551	-1.62227	1.26E-07	3.47E-05	ENSMUSG00000040536	42	15
14804	-7.6551	-1.62227	1.26E-07	3.47E-05	ENSMUSG00000071424	42	8
414069	-7.6551	-1.62227	1.26E-07	3.47E-05	ENSMUSG00000078786	42	11
140919	-7.6551	-1.62227	1.26E-07	3.47E-05	ENSMUSG00000030500	42	14
64011	-7.6551	-1.62227	1.26E-07	3.47E-05	ENSMUSG00000053310	42	12
353130	-2.00476	0.408945	1.46E-07	3.97E-05	ENSMUSG00000049620	121	37
242022	-1.40704	1.950973	1.49E-07	4.02E-05	ENSMUSG00000037016	306	103
545192	-3.24083	-0.79952	1.55E-07	4.13E-05	ENSMUSG00000047507	63	20
68169	-2.45569	-0.24193	1.91E-07	5.03E-05	ENSMUSG00000049001	84	26
243277	-2.29462	-0.12563	2.00E-07	5.24E-05	ENSMUSG00000044017	89	28
72293	-1.85832	0.536649	2.10E-07	5.43E-05	ENSMUSG00000021567	129	41
208890	-1.22496	3.072065	2.18E-07	5.57E-05	ENSMUSG00000040569	632	225
12007	7.709739	-1.74403	2.27E-07	5.57E-05	ENSMUSG00000037053	8	39
100503785	-7.60289	-1.66677	2.27E-07	5.57E-05	NA	41	10
15109	-7.60289	-1.66677	2.27E-07	5.57E-05	ENSMUSG00000020017	41	15
68662	-7.60289	-1.66677	2.27E-07	5.57E-05	ENSMUSG00000064057	41	8
214922	-7.60289	-1.66677	2.27E-07	5.57E-05	ENSMUSG00000072572	41	7
20278	-1.53874	1.34597	2.39E-07	5.79E-05	ENSMUSG00000000216	209	69
75677	-1.3903	1.905936	2.64E-07	6.35E-05	ENSMUSG00000038064	296	101
225266	-2.74161	-0.48407	2.81E-07	6.70E-05	ENSMUSG00000042514	74	23
327956	-1.06514	7.18803	3.11E-07	7.35E-05	ENSMUSG00000020830	10437	3955
170442	-1.38969	1.801697	3.42E-07	8.01E-05	ENSMUSG00000041660	276	95
15366	7.645922	-1.80036	4.11E-07	9.08E-05	ENSMUSG00000020330	11	38
100504211	-7.54873	-1.71274	4.11E-07	9.08E-05	NA	40	14
65256	-7.54873	-1.71274	4.11E-07	9.08E-05	ENSMUSG00000021200	40	16
238690	-7.54873	-1.71274	4.11E-07	9.08E-05	ENSMUSG00000055480	40	11
226245	-7.54873	-1.71274	4.11E-07	9.08E-05	ENSMUSG00000035818	40	4
11444	-7.54873	-1.71274	4.11E-07	9.08E-05	ENSMUSG00000027950	40	5
244694	-7.54873	-1.71274	4.11E-07	9.08E-05	ENSMUSG00000053914	40	16
239096	-2.23664	-0.06351	5.31E-07	0.000116353	ENSMUSG00000059674	92	29
629059	-2.80864	-0.63151	6.97E-07	0.000151535	ENSMUSG00000035184	68	22
100504651	-7.49246	-1.76027	7.45E-07	0.000159382	NA	39	16
56788	-7.49246	-1.76027	7.45E-07	0.000159382	ENSMUSG00000007279	39	6
23985	-1.02845	6.755653	8.19E-07	0.000173926	ENSMUSG00000020651	7673	2985
320452	-1.54821	1.091522	8.72E-07	0.000183821	ENSMUSG00000051048	177	60
14813	-3.79867	-1.2002	8.97E-07	0.000187579	ENSMUSG00000020734	51	18
20377	-1.03657	5.485058	9.70E-07	0.000201317	ENSMUSG00000031548	3192	1241
66607	3.448895	-1.41356	1.28E-06	0.00026285	ENSMUSG00000024678	17	43
435653	1.822543	0.191309	1.32E-06	0.000268853	ENSMUSG00000070524	40	89

100503685	7.509138	-1.91978	1.36E-06	0.000268853	NA	9	36
100502869	-7.4339	-1.80948	1.36E-06	0.000268853	NA	38	4
654812	-7.4339	-1.80948	1.36E-06	0.000268853	ENSMUSG00000028989	38	14
12217	-7.4339	-1.80948	1.36E-06	0.000268853	ENSMUSG00000032589	38	12
12142	-1.91115	0.125816	1.63E-06	0.000320414	ENSMUSG00000038151	100	34
12805	-1.02112	4.99334	1.76E-06	0.000344532	ENSMUSG00000055022	2265	893
20450	1.896215	0.075125	2.02E-06	0.000391199	ENSMUSG00000025789	37	84
244071	-1.71149	0.480084	2.05E-06	0.000394709	ENSMUSG00000025754	122	42
78771	1.963503	-0.06887	2.11E-06	0.00040435	ENSMUSG00000021596	34	78
75568	-4.5165	-1.41447	2.15E-06	0.000408178	ENSMUSG00000039676	46	17
93726	-2.7264	-0.69824	2.32E-06	0.000437208	ENSMUSG00000047222	65	22
20745	1.619678	0.612046	2.35E-06	0.000440359	ENSMUSG00000056222	53	111
381284	-7.37286	-1.86049	2.48E-06	0.000451499	ENSMUSG00000084989	37	6
192190	-7.37286	-1.86049	2.48E-06	0.000451499	ENSMUSG00000038725	37	12
386454	-7.37286	-1.86049	2.48E-06	0.000451499	ENSMUSG00000036492	37	8
12661	-7.37286	-1.86049	2.48E-06	0.000451499	ENSMUSG00000030077	37	14
338368	1.548688	0.731063	2.60E-06	0.000471864	ENSMUSG00000049687	58	118
12447	-2.05478	-0.10526	2.89E-06	0.000519307	ENSMUSG00000002068	88	30
72500	-1.52957	0.872863	2.90E-06	0.000519307	ENSMUSG00000089762	153	54
94111	-2.98448	-1.01015	3.55E-06	0.000631034	ENSMUSG00000053863	55	20
12560	-1.7817	0.302562	3.72E-06	0.00065171	ENSMUSG00000061048	110	38
58869	-1.59848	0.639926	3.87E-06	0.00067373	ENSMUSG00000027674	133	47
246049	1.674239	0.372893	4.00E-06	0.000691879	ENSMUSG00000020264	46	97
432628	-1.67091	0.449916	4.25E-06	0.000725827	ENSMUSG00000037336	119	42
19734	-1.65712	0.439714	4.25E-06	0.000725827	ENSMUSG00000026475	118	42
18190	-7.30912	-1.91344	4.54E-06	0.000761323	ENSMUSG00000033768	36	15
433700	-7.30912	-1.91344	4.54E-06	0.000761323	ENSMUSG00000066196	36	13
22116	-7.30912	-1.91344	4.54E-06	0.000761323	ENSMUSG00000059891	36	12
594844	-1.61979	0.563146	4.66E-06	0.000776716	ENSMUSG00000044550	127	45
24131	3.708012	-1.69978	4.81E-06	0.000797471	ENSMUSG00000021798	16	38
338346	-4.42381	-1.4922	6.10E-06	0.000999344	ENSMUSG00000053164	44	17
78547	-4.42381	-1.4922	6.10E-06	0.000999344	ENSMUSG00000044997	44	17

269152	-1.3129	1.404496	6.21E-06	0.001010167	ENSMUSG00000026494	209	78
14169	-2.54635	-0.84208	6.24E-06	0.001010167	ENSMUSG00000025551	59	22
13869	-3.63427	-1.33655	6.44E-06	0.001036114	ENSMUSG00000062209	47	18
100503002	-2.20958	-0.43633	6.58E-06	0.001052604	NA	73	26
263803	-1.29424	1.50083	6.95E-06	0.001104693	ENSMUSG00000026785	222	83
67503	-2.37324	-0.60342	7.29E-06	0.001152417	ENSMUSG00000103746	67	24
20379	1.867465	-0.14144	7.35E-06	0.001154313	ENSMUSG00000021319	34	74
320046	-2.70497	-0.95069	7.46E-06	0.001164998	ENSMUSG00000052125	56	21
320064	-1.54068	0.641726	7.57E-06	0.001175045	NA	132	48
11941	-1.01507	3.579308	7.63E-06	0.001178635	ENSMUSG00000030302	855	345
216454	7.27607	-2.11884	8.35E-06	0.001194614	ENSMUSG00000074639	9	33
622124	7.27607	-2.11884	8.35E-06	0.001194614	ENSMUSG00000104401	14	33
246177	-7.24244	-1.96848	8.35E-06	0.001194614	ENSMUSG00000020437	35	2
667631	-7.24244	-1.96848	8.35E-06	0.001194614	NA	35	9
100504549	-7.24244	-1.96848	8.35E-06	0.001194614	NA	35	15
219144	-7.24244	-1.96848	8.35E-06	0.001194614	ENSMUSG00000043157	35	14
415115	-7.24244	-1.96848	8.35E-06	0.001194614	ENSMUSG00000039873	35	16
58203	-7.24244	-1.96848	8.35E-06	0.001194614	ENSMUSG00000027514	35	13
76072	-7.24244	-1.96848	8.35E-06	0.001194614	ENSMUSG00000063851	35	8
208628	-7.24244	-1.96848	8.35E-06	0.001194614	ENSMUSG00000029414	35	5
100041450	-7.24244	-1.96848	8.35E-06	0.001194614	NA	35	14
18752	-7.24244	-1.96848	8.35E-06	0.001194614	ENSMUSG00000078816	35	11
78052	-7.24244	-1.96848	8.35E-06	0.001194614	ENSMUSG00000013091	35	12
73619	-7.24244	-1.96848	8.35E-06	0.001194614	NA	35	11
55983	-1.27329	1.486141	9.20E-06	0.001308453	ENSMUSG00000035357	219	83
11808	-1.0469	2.936337	9.48E-06	0.001341291	ENSMUSG00000032080	555	223
21940	-4.37513	-1.53276	1.03E-05	0.001451825	ENSMUSG00000030336	43	17
68162	-1.43941	0.843924	1.16E-05	0.001612979	NA	148	55
77974	1.505371	0.564984	1.16E-05	0.001612979	ENSMUSG00000021123	54	106
227580	-1.23818	1.543576	1.16E-05	0.001612979	ENSMUSG00000049630	226	87
234857	-1.45986	0.70782	1.17E-05	0.001612979	ENSMUSG00000010154	136	51
232785	-1.20812	1.67379	1.20E-05	0.001652729	ENSMUSG00000072653	245	95

330941	1.29518	1.210631	1.23E-05	0.001682882	ENSMUSG00000078307	84	152
71724	2.186982	-0.72051	1.35E-05	0.001831346	ENSMUSG00000064294	25	56
238988	-2.31789	-0.64674	1.54E-05	0.001977971	ENSMUSG00000040640	65	24
330277	2.133159	-0.64349	1.54E-05	0.001977971	ENSMUSG00000039742	26	58
108760	-7.17253	-2.02578	1.54E-05	0.001977971	ENSMUSG00000021130	34	0
18095	-7.17253	-2.02578	1.54E-05	0.001977971	ENSMUSG00000022061	34	14
210573	-7.17253	-2.02578	1.54E-05	0.001977971	ENSMUSG00000096847	34	16
100504187	-7.17253	-2.02578	1.54E-05	0.001977971	NA	34	8
64406	-7.17253	-2.02578	1.54E-05	0.001977971	ENSMUSG00000075304	34	6
384214	-7.17253	-2.02578	1.54E-05	0.001977971	ENSMUSG00000033805	34	7
109163	-7.17253	-2.02578	1.54E-05	0.001977971	ENSMUSG00000085355	34	14
11814	-7.17253	-2.02578	1.54E-05	0.001977971	ENSMUSG00000032081	34	14
74100	-7.17253	-2.02578	1.54E-05	0.001977971	ENSMUSG00000032503	34	9
30878	-7.17253	-2.02578	1.54E-05	0.001977971	ENSMUSG00000037010	34	12
72804	-3.11757	-1.26252	1.58E-05	0.002015205	NA	48	19
100503922	-3.54446	-1.41007	1.73E-05	0.00218221	NA	45	18
667373	-3.54446	-1.41007	1.73E-05	0.00218221	ENSMUSG00000079339	45	18
18682	-3.54446	-1.41007	1.73E-05	0.00218221	ENSMUSG00000025537	45	18
20717	-4.32475	-1.57453	1.74E-05	0.002183468	ENSMUSG00000079012	42	17
18793	-1.51914	0.536599	1.81E-05	0.002255621	ENSMUSG00000046223	123	46
100126229	1.790993	-0.19828	1.86E-05	0.002310137	ENSMUSG00000085105	34	71
53814	-2.79753	-1.16001	2.14E-05	0.002646205	ENSMUSG00000028141	50	20
320798	-1.97337	-0.37464	2.19E-05	0.002697783	NA	74	28
75019	1.613977	0.141382	2.23E-05	0.002724953	ENSMUSG00000021872	42	84
23886	-1.36746	0.862461	2.44E-05	0.002973498	ENSMUSG00000038508	148	57
319336	-3.07468	-1.29696	2.53E-05	0.003061251	NA	47	19
13417	-1.9117	-0.21447	2.53E-05	0.003061251	ENSMUSG00000033826	81	30
232370	-1.10839	1.981838	2.60E-05	0.003122404	ENSMUSG00000008153	295	119
17175	-2.59842	-1.03499	2.71E-05	0.003174414	ENSMUSG00000028979	53	21
100038371	7.189189	-2.19152	2.86E-05	0.003174414	NA	11	32
118452	7.189189	-2.19152	2.86E-05	0.003174414	ENSMUSG00000022296	12	32
27412	7.189189	-2.19152	2.86E-05	0.003174414	ENSMUSG00000070526	4	32

18054	7.189189	-2.19152	2.86E-05	0.003174414	ENSMUSG00000032484	8	32
78185	7.189189	-2.19152	2.86E-05	0.003174414	ENSMUSG00000039545	8	32
76184	-7.09905	-2.08554	2.86E-05	0.003174414	ENSMUSG00000044749	33	4
27386	-7.09905	-2.08554	2.86E-05	0.003174414	ENSMUSG00000021010	33	10
100504637	-7.09905	-2.08554	2.86E-05	0.003174414	ENSMUSG00000100199	33	6
67849	-7.09905	-2.08554	2.86E-05	0.003174414	ENSMUSG00000024791	33	12
227717	-7.09905	-2.08554	2.86E-05	0.003174414	ENSMUSG00000043102	33	12
100504361	-7.09905	-2.08554	2.86E-05	0.003174414	NA	33	11
12561	-7.09905	-2.08554	2.86E-05	0.003174414	ENSMUSG00000000305	33	6
215090	-7.09905	-2.08554	2.86E-05	0.003174414	ENSMUSG00000042763	33	16
329973	-7.09905	-2.08554	2.86E-05	0.003174414	NA	33	4
78263	-7.09905	-2.08554	2.86E-05	0.003174414	NA	33	11
69665	-7.09905	-2.08554	2.86E-05	0.003174414	ENSMUSG00000006143	33	14
434341	-7.09905	-2.08554	2.86E-05	0.003174414	ENSMUSG00000074151	33	11
26927	-7.09905	-2.08554	2.86E-05	0.003174414	ENSMUSG00000050397	33	12
170758	-1.5418	0.355321	2.92E-05	0.003220997	ENSMUSG00000018012	110	42
12291	-4.21839	-1.66202	2.95E-05	0.003233831	ENSMUSG00000020866	40	17
665610	-4.21839	-1.66202	2.95E-05	0.003233831	NA	40	17
71818	-2.41249	-0.94694	3.27E-05	0.003569582	ENSMUSG00000113434	55	22
103098	-1.10735	1.866275	3.34E-05	0.003633373	ENSMUSG00000019894	273	111
195727	-2.75705	-1.19202	3.36E-05	0.003633373	ENSMUSG00000059493	49	20
73902	1.578863	0.065226	3.77E-05	0.004070391	ENSMUSG00000072423	41	80
58243	-3.03048	-1.33226	4.05E-05	0.004330684	ENSMUSG00000055430	46	19
105833	2.683599	-1.37913	4.05E-05	0.004330684	ENSMUSG00000003354	19	42
66968	2.462184	-1.11302	4.17E-05	0.00444483	ENSMUSG00000011305	21	47
381306	1.243778	1.047619	4.22E-05	0.004483749	ENSMUSG00000041406	78	136
382384	-1.29555	0.972774	4.51E-05	0.004749252	ENSMUSG00000035963	157	62
320277	-3.97878	-1.85546	4.58E-05	0.004786754	ENSMUSG00000072663	36	17
67580	3.420105	-1.93046	4.58E-05	0.004786754	ENSMUSG00000041673	16	34
668041	-3.44869	-1.48769	4.68E-05	0.004843444	NA	43	18
100503912	-3.44869	-1.48769	4.68E-05	0.004843444	NA	43	18
320878	-1.52109	0.286354	4.75E-05	0.004843444	ENSMUSG00000038244	105	41

73989	-1.01262	2.384755	4.93E-05	0.004843444	ENSMUSG00000115422	379	159
68659	-1.61941	0.051981	4.94E-05	0.004843444	ENSMUSG00000027955	92	36
72184	-1.61941	0.051981	4.94E-05	0.004843444	ENSMUSG00000035298	92	36
100504639	-4.16211	-1.70792	5.00E-05	0.004843444	NA	39	17
14561	-2.71539	-1.22478	5.25E-05	0.004843444	ENSMUSG00000025352	48	20
212712	-7.02164	-2.14798	5.33E-05	0.004843444	ENSMUSG00000038331	32	10
19886	-7.02164	-2.14798	5.33E-05	0.004843444	ENSMUSG00000019893	32	2
17001	-7.02164	-2.14798	5.33E-05	0.004843444	ENSMUSG00000020377	32	12
12569	-7.02164	-2.14798	5.33E-05	0.004843444	ENSMUSG00000048895	32	10
214321	-7.02164	-2.14798	5.33E-05	0.004843444	ENSMUSG00000072974	32	8
13488	-7.02164	-2.14798	5.33E-05	0.004843444	ENSMUSG00000021478	32	8
380930	-7.02164	-2.14798	5.33E-05	0.004843444	ENSMUSG00000115149	32	13
116838	-7.02164	-2.14798	5.33E-05	0.004843444	ENSMUSG00000037386	32	13
100503680	-7.02164	-2.14798	5.33E-05	0.004843444	NA	32	10
12448	-7.02164	-2.14798	5.33E-05	0.004843444	ENSMUSG00000028212	32	13
14191	-7.02164	-2.14798	5.33E-05	0.004843444	ENSMUSG00000028874	32	12
71176	-7.02164	-2.14798	5.33E-05	0.004843444	ENSMUSG00000089984	32	16
21789	-7.02164	-2.14798	5.33E-05	0.004843444	ENSMUSG00000029664	32	4
100503974	-7.02164	-2.14798	5.33E-05	0.004843444	NA	32	12
80720	-7.02164	-2.14798	5.33E-05	0.004843444	ENSMUSG00000031860	32	12
102920	-7.02164	-2.14798	5.33E-05	0.004843444	ENSMUSG00000031262	32	8
71860	-6.93983	-2.21335	5.33E-05	0.004843444	ENSMUSG00000020904	31	7
432396	-6.93983	-2.21335	5.33E-05	0.004843444	ENSMUSG00000086191	31	6
100862206	-6.93983	-2.21335	5.33E-05	0.004843444	ENSMUSG00000113280	31	14
100504566	-6.93983	-2.21335	5.33E-05	0.004843444	NA	31	7
271278	-6.93983	-2.21335	5.33E-05	0.004843444	ENSMUSG00000044361	31	16
100502943	-6.93983	-2.21335	5.33E-05	0.004843444	NA	31	12
329078	-6.93983	-2.21335	5.33E-05	0.004843444	ENSMUSG00000087095	31	13
14810	-6.93983	-2.21335	5.33E-05	0.004843444	ENSMUSG00000026959	31	10
12386	-6.93983	-2.21335	5.33E-05	0.004843444	ENSMUSG00000063063	31	13
232966	-6.93983	-2.21335	5.33E-05	0.004843444	ENSMUSG00000068962	31	12
243897	-6.93983	-2.21335	5.33E-05	0.004843444	ENSMUSG00000031493	31	8

11937	-6.93983	-2.21335	5.33E-05	0.004843444	ENSMUSG00000030730	31	16
11604	-6.93983	-2.21335	5.33E-05	0.004843444	ENSMUSG00000005705	31	4
245446	-6.93983	-2.21335	5.33E-05	0.004843444	ENSMUSG00000046699	31	2
80903	-6.93983	-2.21335	5.33E-05	0.004843444	ENSMUSG00000031230	31	2
76257	-1.235	1.075288	5.62E-05	0.005092847	ENSMUSG00000010064	166	67
13123	-1.4725	0.357414	5.85E-05	0.005284496	ENSMUSG00000039519	109	43
64085	-1.50517	0.274892	6.04E-05	0.005438057	ENSMUSG00000032452	104	41
654318	1.106598	1.561708	6.64E-05	0.005957632	ENSMUSG00000085408	112	183
76670	-1.4235	0.467958	6.78E-05	0.006060251	ENSMUSG00000039543	116	46
16669	-1.01096	2.258667	7.26E-05	0.006469395	ENSMUSG00000020911	348	147
224419	2.318713	-1.04685	7.46E-05	0.006623612	ENSMUSG00000025610	22	48
100502619	-3.34611	-1.5699	7.68E-05	0.006781059	NA	41	18
628870	-1.01323	2.147043	7.68E-05	0.006781059	ENSMUSG00000091455	323	137
320004	-3.9121	-1.90834	8.08E-05	0.007107383	NA	35	17
246133	-1.2165	1.08976	8.66E-05	0.007588477	ENSMUSG00000039672	167	68
12506	-1.09791	1.59843	8.72E-05	0.007624637	ENSMUSG00000015355	228	95
74665	1.024119	1.943442	8.83E-05	0.007692283	ENSMUSG00000056598	147	230
52882	-2.26231	-0.86394	9.19E-05	0.007983533	ENSMUSG00000021719	57	23
28240	-2.44289	-1.15596	9.83E-05	0.008204501	ENSMUSG00000009292	49	21
215456	1.248955	0.729089	9.84E-05	0.008204501	ENSMUSG00000046338	65	112
213043	-6.8531	-2.28194	9.96E-05	0.008204501	ENSMUSG00000079554	30	12
16524	-6.8531	-2.28194	9.96E-05	0.008204501	ENSMUSG00000038026	30	7
16545	-6.8531	-2.28194	9.96E-05	0.008204501	ENSMUSG00000019932	30	16
432530	-6.8531	-2.28194	9.96E-05	0.008204501	ENSMUSG00000020431	30	6
217143	-6.8531	-2.28194	9.96E-05	0.008204501	ENSMUSG00000070337	30	16
21819	-6.8531	-2.28194	9.96E-05	0.008204501	ENSMUSG00000053469	30	10
29870	-6.8531	-2.28194	9.96E-05	0.008204501	ENSMUSG00000022385	30	8
668225	-6.8531	-2.28194	9.96E-05	0.008204501	ENSMUSG00000095440	30	16
68230	-6.8531	-2.28194	9.96E-05	0.008204501	ENSMUSG00000116852	30	12
332396	-6.8531	-2.28194	9.96E-05	0.008204501	ENSMUSG00000040901	30	2
623781	-6.8531	-2.28194	9.96E-05	0.008204501	ENSMUSG00000055926	30	11
18389	-6.8531	-2.28194	9.96E-05	0.008204501	ENSMUSG00000027584	30	12

11489	-6.8531	-2.28194	9.96E-05	0.008204501	ENSMUSG00000054555	30	6
75770	-6.8531	-2.28194	9.96E-05	0.008204501	ENSMUSG00000053046	30	12
80901	-6.8531	-2.28194	9.96E-05	0.008204501	ENSMUSG00000048521	30	14
